# PTP1B inhibitors protect against acute lung injury and regulate CXCR4 signaling in neutrophils

**DOI:** 10.1172/jci.insight.158199

**Published:** 2022-07-22

**Authors:** Dongyan Song, Jose M. Adrover, Christy Felice, Lisa N. Christensen, Xue-Yan He, Joseph R. Merrill, John E. Wilkinson, Tobias Janowitz, Scott K. Lyons, Mikala Egeblad, Nicholas K. Tonks

**Affiliations:** 1 Cold Spring Harbor Laboratory, Cold Spring Harbor, New York, USA.; 2 Molecular and Cellular Biology Graduate Program, Stony Brook University, Stony Brook, New York, USA.; 3 Unit for Laboratory Animal Medicine, Department of Pathology, University of Michigan, Ann Arbor, Michigan, USA.

**Keywords:** Cell Biology, Neutrophils

## Abstract

Acute lung injury (ALI) can cause acute respiratory distress syndrome (ARDS), a lethal condition with limited treatment options and currently a common global cause of death due to COVID-19. ARDS secondary to transfusion-related ALI (TRALI) has been recapitulated preclinically by anti–MHC-I antibody administration to LPS-primed mice. In this model, we demonstrate that inhibitors of PTP1B, a protein tyrosine phosphatase that regulates signaling pathways of fundamental importance to homeostasis and inflammation, prevented lung injury and increased survival. Treatment with PTP1B inhibitors attenuated the aberrant neutrophil function that drives ALI and was associated with release of myeloperoxidase, suppression of neutrophil extracellular trap (NET) formation, and inhibition of neutrophil migration. Mechanistically, reduced signaling through the CXCR4 chemokine receptor, particularly to the activation of PI3Kγ/AKT/mTOR, was essential for these effects, linking PTP1B inhibition to promoting an aged-neutrophil phenotype. Considering that dysregulated activation of neutrophils has been implicated in sepsis and causes collateral tissue damage, we demonstrate that PTP1B inhibitors improved survival and ameliorated lung injury in an LPS-induced sepsis model and improved survival in the cecal ligation and puncture–induced (CLP-induced) sepsis model. Our data highlight the potential for PTP1B inhibition to prevent ALI and ARDS from multiple etiologies.

## Graphical abstract

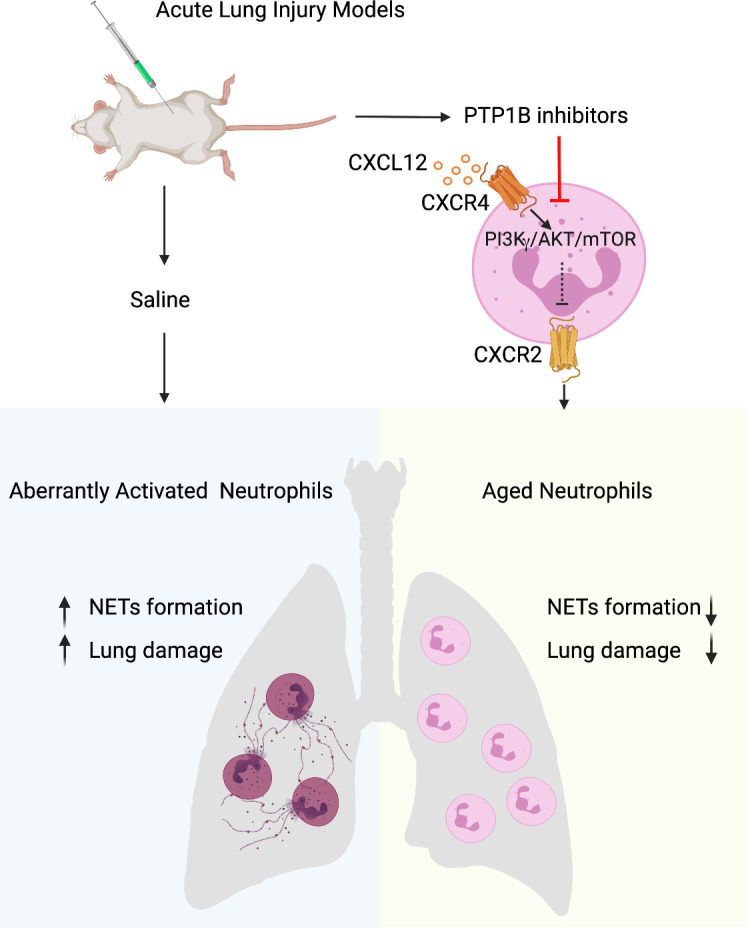

## Introduction

Acute respiratory distress syndrome (ARDS) is a life-threatening clinical condition that often requires ventilation and management in intensive care. ARDS is a consequence of acute lung injury (ALI), which may arise from diverse underlying causes, including viral or bacterial pneumonia, sepsis, trauma, or transfusion-related ALI (TRALI) (1–3). The poor prognosis of ARDS is attributable in part to the limited understanding of the pathogenic mechanisms of ALI, which results in few treatment options and has created an urgent unmet medical need.

The aberrant activation of neutrophils is one of the hallmarks of ALI (4). Neutrophils are the primary effector cells in the innate immune response, providing protection from invading pathogens (5). These beneficial antimicrobial functions are balanced with potentially deleterious inflammatory effects and, therefore, are tightly regulated through control of degranulation, reactive oxygen species (ROS) production, NET formation, and lifespan in circulation. The neutrophil circulatory half-life is less than 1 day in humans, requiring constant replenishment from BM (6). The phenotypic and functional alterations starting from neutrophil release to clearance exhibit a circadian pattern, which has been described as neutrophil aging (7). Recently, a cell-intrinsic program was reported to underlie neutrophil aging and has been linked to disease. The molecular clock brain and muscle arnt-like protein-1 (BMAL1) controls the expression of CXCL2, a chemokine that activates CXCR2, to promote neutrophil aging, whereas CXCR4 antagonizes CXCR2 signaling (8). Neutrophil aging was highlighted as a “programmed disarming,” which decreased neutrophil capacity to inflict damage after infiltrating target tissues, such as the lung (9), and diminished the risk for damage to the vascular system (8, 10). It was proposed that therapeutic intervention to regulate neutrophil aging may protect tissues from damaging, hyperinflammatory states (9); however, the pathways downstream of the CXCR2/CXCR4 axis remain elusive.

Protein tyrosine phosphatase-1B (PTP1B, encoded by *Ptpn1*) regulates signaling events that are of fundamental importance to homeostatic control (11) and has been implicated in the regulation of adaptive and innate immune cell signaling (12). Specific deletion of *Ptpn1* in B cells results in systemic autoimmunity with increased B cell numbers and total IgG concentration. In B cells, PTP1B negatively regulates CD40 and B cell activating factor receptor (BAFF-R) signaling, which promotes production of high-affinity IgG antibodies and B cell survival, respectively (13). Studies regarding the function of PTP1B in macrophages have been controversial. During myeloid lineage development, loss of PTP1B leads to increased monocyte/macrophage population and macrophage activity through enhancing the tyrosine phosphorylation of macrophage colony-stimulating factor 1 receptor (CSF-1R) (14). Nevertheless, inhibition of PTP1B has also been shown to induce M2 (antiinflammatory) macrophage polarization (15) and increase IL-10–mediated TYK2/STAT3 antiinflammatory signaling (16). Moreover, myeloid lineage depletion of PTP1B in mice results in reduced inflammation and protection against LPS-induced endotoxemia (17) and in impaired DC maturation and migration (18), which suggests that the function of PTP1B is dependent on cellular context and stimuli.

In neutrophils, which are the most abundant myeloid cells, the function of PTP1B remains largely uncharacterized. In this study, we report the effects of 2 distinct, allosteric inhibitors of PTP1B that are drug candidates. MSI-1436 has been shown to engage PTP1B as a target in cell and animal models of breast cancer (19) and has been tested in clinical trials for obesity and metastatic breast cancer. DPM-1003 differs from MSI-1436 in that the charged sulfate group and spermine tail are replaced by uncharged ester and pyridine ring–containing moieties. Both compounds were examined for their effects on neutrophil phenotypic plasticity, as well as the induction of ALI in murine models of TRALI and LPS-induced sepsis. We reveal a beneficial effect in these clinically relevant mouse models of lung inflammation, in which inhibition of PTP1B prevents TRALI and ameliorates LPS-induced sepsis. In addition, we demonstrate enhanced survival in the cecal ligation and puncture–induced (CLP-induced) sepsis model. The similarities between the 2 compounds in their protective effects highlight the importance of their function as PTP1B inhibitors. Furthermore, we present a mechanistic link between PTP1B inhibition and modulation of an aged-neutrophil phenotype, involving PI3Kγ/AKT/mTOR-dependent suppression of CXCR4 function. These data highlight PTP1B as a potential therapeutic target for ARDS.

## Results

### PTP1B inhibitors improved survival and ameliorated lung damage in the TRALI mouse model.

**Figure 1:**
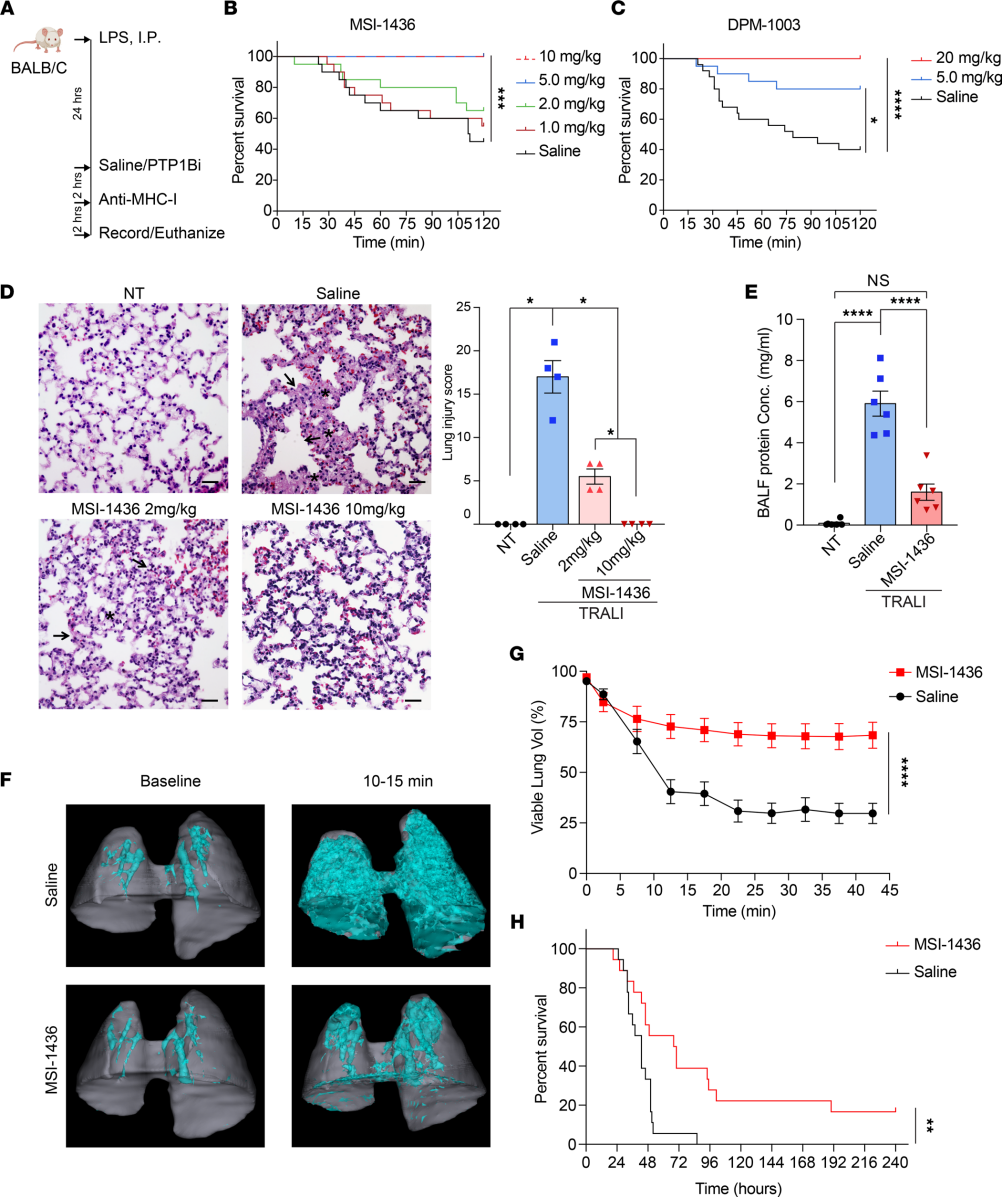
PTP1B inhibitors improved survival and ameliorated lung damage in the TRALI mouse model. (**A**) Schematic illustration of the TRALI induction and PTP1B inhibition protocol. (**B**) The survival curves of TRALI mice treated with increasing concentrations of the PTP1B inhibitor MSI-1436 or saline (*n* = 20 mice, 2 independent biological repeats). (**C**) The survival curves of TRALI mice treated with increasing concentrations of the PTP1B inhibitor DPM-1003 or saline (*n* = 20–25 mice, 2 independent biological repeats). (**D**) Representative images of H&E-stained lung tissue from no treatment mice (NT), and TRALI mice after administering saline, MSI-1436 at 2 mg/kg, or MSI-1436 at 10 mg/kg. Arrows indicate alveolar damage. Asterisks indicate edema or hyaline membranes. Scale bars: 25 μm. Lung injury scores for each treatment group. (*n* = 4 mice per group). (**E**) The protein concentrations in the bronchoalveolar lavage fluid (BALF) collected from NT or TRALI mice treated with either saline or 10 mg/kg MSI-1436 (*n* = 6 mice per group). (**F**) Representative 3D-rendered images of lung volumes from CT scans of mice from the saline- and MSI-1436–treated TRALI mice. Cyan represents hyperdense areas of edema and vasculature (Hounsfield units [HU] > 0); gray represents hypodense regions of airspace — i.e. viable lung (HU < 0). (**G**) The percentage of viable lung volume (volume of viable lung/volume of total lung) calculated from longitudinal CT scans in 2 experimental groups as in **F**. (*n* = 7–8 mice per group). (**H**) The survival curves of CLP-induced sepsis model treated either with saline or 5 mg/kg MSI-1436 at 2 hours before surgery (*n* = 18 mice per group). Data are presented as mean ± SEM. Statistical analysis for **B**, **C**, and **H** by was done log-rank (Mantel-Cox) test; by nonparametric 2-tailed Mann-Whitney test for **D**; by 1-way ANOVA with Tukey’s multiple-comparison test for **E**; and by 2-way ANOVA for **G**. *P < 0.05, **P < 0.01, ****P < 0.0001

To investigate the function of PTP1B in the biology of ALI/ARDS — in particular, its function in neutrophils — we examined the effect of PTP1B inhibitors in murine disease models that were driven by aberrant activation of neutrophils. First, we tested PTP1B inhibitors in an established TRALI model, summarized in Figure 1A, in which neutrophils and neutrophil extracellular traps (NETs) play a crucial role (20, 21). Following pretreatment with LPS as a priming step, we tested the effects of injecting single doses of PTP1B inhibitors, i.p., 2 hours prior to i.v. injection of anti–major histocompatibility complex-I (anti–MHC-I) antibodies. Timing and route of the injection were chosen so that the maximal dose of the compound in serum coincided with the initiation of the acute phase of lung injury. We tested the effect of MSI-1436 (Supplemental Figure 1A; supplemental material available online with this article; https://doi.org/10.1172/jci.insight.158199DS1), an allosteric inhibitor of PTP1B for which efficacy and specificity has been demonstrated in animal models of HER2^+^ breast cancer (19). Compared with the saline control group, MSI-1436 treatment resulted in a dose-dependent increase in survival. At doses of 5 and 10 mg/kg, MSI-1436 resulted in 100% survival of mice at the 2-hour time point compared with 40% survival of mice in the control group (Figure 1B). Similar dose-dependent protective effects were observed following treatment with DPM-1003, a structurally distinct inhibitor of PTP1B compared with MSI-1436 (Figure 1C and Supplemental Figure 1B).

We compared the histopathological appearance of lung tissue taken from mice in which TRALI had not been induced (no-treatment [NT]controls) with TRALI mice treated with saline, or with different doses of MSI-1436. These analyses revealed marked accumulation of alveolar edema and hyaline membranes following vehicle (saline) treatment of the TRALI mice, whereas after treatment with MSI-1436 at 10 mg/kg, the lungs appeared nearly normal (Figure 1D). The 2 mg/kg MSI-1436–treated group also displayed reduced edema and hyaline membranes in the alveolar space. To quantify lung damage, we defined a lung injury score that reflected a combination of gross examination of damage distribution (multifocal, locally extensive, diffuse), accumulation of edema and hyaline membrane, and alveolar and vessel damage (Figure 1D). Consistent with the survival data, treatment with MSI-1436 at 2 mg/kg improved the lung injury score reflecting moderate injury, whereas 10 mg/kg–treated mice did not present signs of lung injury. To assess pulmonary permeability, we measured the protein leakage in the bronchoalveolar lavage fluid (BALF) and edema formation. In the saline-treated TRALI mice, the BALF protein concentration significantly increased compared with NT controls, whereas in the MSI-1436-treated TRALI mice, the levels were not significantly elevated compared with the control group (Figure 1E). We used longitudinal CT scans to monitor the accumulation of edema after TRALI induction in the mice pretreated with either saline or MSI-1436; viable lung airspace was defined as lung volume not occupied by edema. After anti–MHC-I antibody injection, there is an acute increase of pulmonary edema (Figure 1F and Supplemental Figure 1C); however, MSI-1436 treatment prevented progressive loss of viable lung volume compared with saline treatment (Figure 1, F and G; Supplemental Figure 1C; and Supplemental Videos 1 and 2). Overall, these observations demonstrate that PTP1B inhibitors are protective against lung damage, consistent with promoting survival in the TRALI model.

### Treatment with PTP1B inhibitors improved survival in additional models of sepsis.

To generalize this observation, we examined the effect of PTP1B inhibitors, administered as a single dose, in the CLP-induced polymicrobial sepsis and LPS-induced lethal endotoxemia (sepsis) models, which recapitulate the systemic inflammation and shock-like state of bacterial septic challenge (22). The high lethality of sepsis is associated with dysregulation of the host inflammatory response, including the detrimental effects of aberrant activation of neutrophils (23–27). In the CLP sepsis model, which is a clinically relevant experimental model of septic peritonitis in humans, the cecum was ligated and perforated to cause drainage of cecal bacteria into peritoneal cavity (28). We administered either saline or 5 mg/kg MSI-1436, 2 hours before surgery. The saline-treated mice all died within 96 hours after CLP surgery, whereas ~20% of mice were alive at day 10 in the MSI-1436–pretreated group (Figure 1H). In addition, we treated the mice with either saline or 5 mg/kg MSI-1436, 6 hours after CLP surgery, which is the time at which we observed onset of symptoms, such as reduced activity and lethargy. Treatment with MSI-1436 after surgery did not exacerbate the symptoms, and did not significantly delay death (Supplemental 1D).

In an additional model, we pretreated with either saline or 10 mg/kg MSI-1436 for 2 hours and then injected LPS i.p. at 2 concentrations to induce sepsis. The high concentration of LPS (30 mg/kg) led to 100% lethality within 24 hours, whereas MSI-1436 pretreatment significantly prolonged the survival time and increased the survival rate to 20% (Supplemental Figure 1, C and D). Following administration of LPS at 15 mg/kg, all the mice died within 50 hours in the saline control group; in contrast, 20% of the mice survived, and the death of the remaining mice was substantially delayed in the MSI-1436–treated group (Supplemental Figure 1, E–G). Consistently, 24 hours after 15 mg/kg LPS challenge, we observed that MSI-1436–treated mice displayed less pulmonary edema than the saline control (Supplemental Figure 1H). Taken together, PTP1B inhibitors have a prophylactic function to improve survival in TRALI and CLP- and LPS-induced sepsis models. In light of the complicated inflammatory response involved in the sepsis models, we focused on the neutrophil-dependent TRALI model (20, 29, 30) for mechanistic analyses.

### Treatment with PTP1B inhibitors induced neutrophilia.

**Figure 2:**
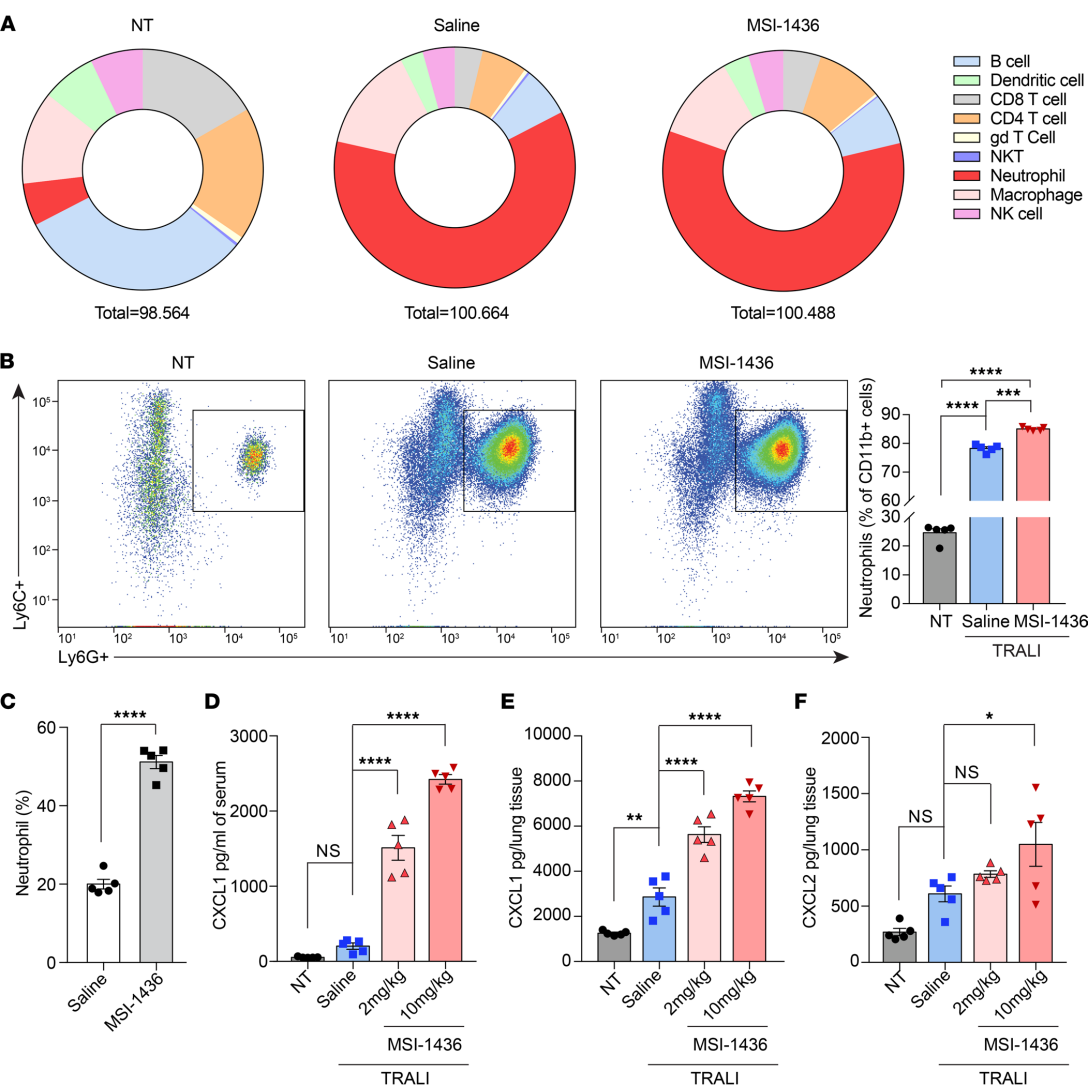
Treatment with PTP1B inhibitors in vivo induced neutrophilia. (**A**) Pie charts of flow cytometry analysis to measure infiltration of 9 immune cell populations into lung tissues. The numbers are average abundance of each immune cell subset (% of CD45^+^ cells, *n* = 5). For saline- and MSI-1436–treated (10 mg/kg) groups, the lungs were harvested 30 minutes after TRALI induction. (**B**) Representative flow cytometry plots for neutrophils infiltrated into lung tissues. Quantification of percentage of neutrophil population out of myeloid cells. (**C**) Percentage of neutrophils (relative to total WBCs) in peripheral blood from mice treated with saline or 10 mg/kg MSI-1436 for 2.5 hours (*n* = 5). (**D** and **E**) CXCL1 levels in serum (**D**) and matched lung tissue (**E**) from NT and TRALI mice treated with saline or MSI-1436 at the indicated doses (*n* = 5). (**F**) CXCL2 levels in lung tissue from NT and TRALI mice treated with saline or MSI-1436 at the indicated doses (*n* = 5). Data are presented as mean ± SEM. Statistical analysis for **C** was performed by 2-tailed Student’s *t* test and by 1-way ANOVA with Tukey’s multiple-comparison test for **B** and **D**–**F**. *P < 0.05, **P < 0.01, ***P < 0.001, ****P < 0.0001.

We profiled the accumulation of immune cells in lungs and the circulation 30 minutes after anti–MHC-I antibody injection. In agreement with reports that neutrophils are critical for the initiation of TRALI (21, 30–32), the most dramatic increase we observed was in neutrophil numbers (Figure 2A and Supplemental Figure 2, A and B). Unexpectedly, we observed that, following pretreatment with MSI-1436, CD11b^+^Ly6C^+^Ly6G^+^ neutrophil infiltration into lung tissues after TRALI induction was elevated compared with saline-treated mice (Figure 2B). This increase of pulmonary neutrophil accumulation prompted us to examine directly the effect of PTP1B inhibitors on neutrophils. We examined hematological parameters after treatment with PTP1B inhibitors and observed that the number of neutrophils in the peripheral blood increased ~3-fold following administration of either MSI-1436 or DPM-1003 (Figure 2C, Supplemental Figure 2C, and Supplemental Table 1).

To examine the mechanism underlying the neutrophilia that occurred following treatment with PTP1B inhibitors, we analyzed cytokine arrays for both serum and lung tissue in the no-treatment control and TRALI mice, injected with saline or MSI-1436; the results were then validated by ELISA (Supplemental Figure 3, A and B). Upon MSI-1436 treatment, the level of CXCL1 markedly increased in both serum and lung homogenates, and the level of CXCL2 was elevated in lung homogenates (Figure 2, D–F). Furthermore, we observed that the chemokine CXCL1 was elevated in plasma in response to both MSI-1436 and DPM-1003, independently of TRALI induction (Supplemental Figure 3, C and D). Consistent with our observation of neutrophilia, CXCL1 and CXCL2 are principal chemotactic cues for neutrophil migration (33).

### Treatment with PTP1B inhibitors induced an aged-neutrophil phenotype in vivo.

**Figure 3:**
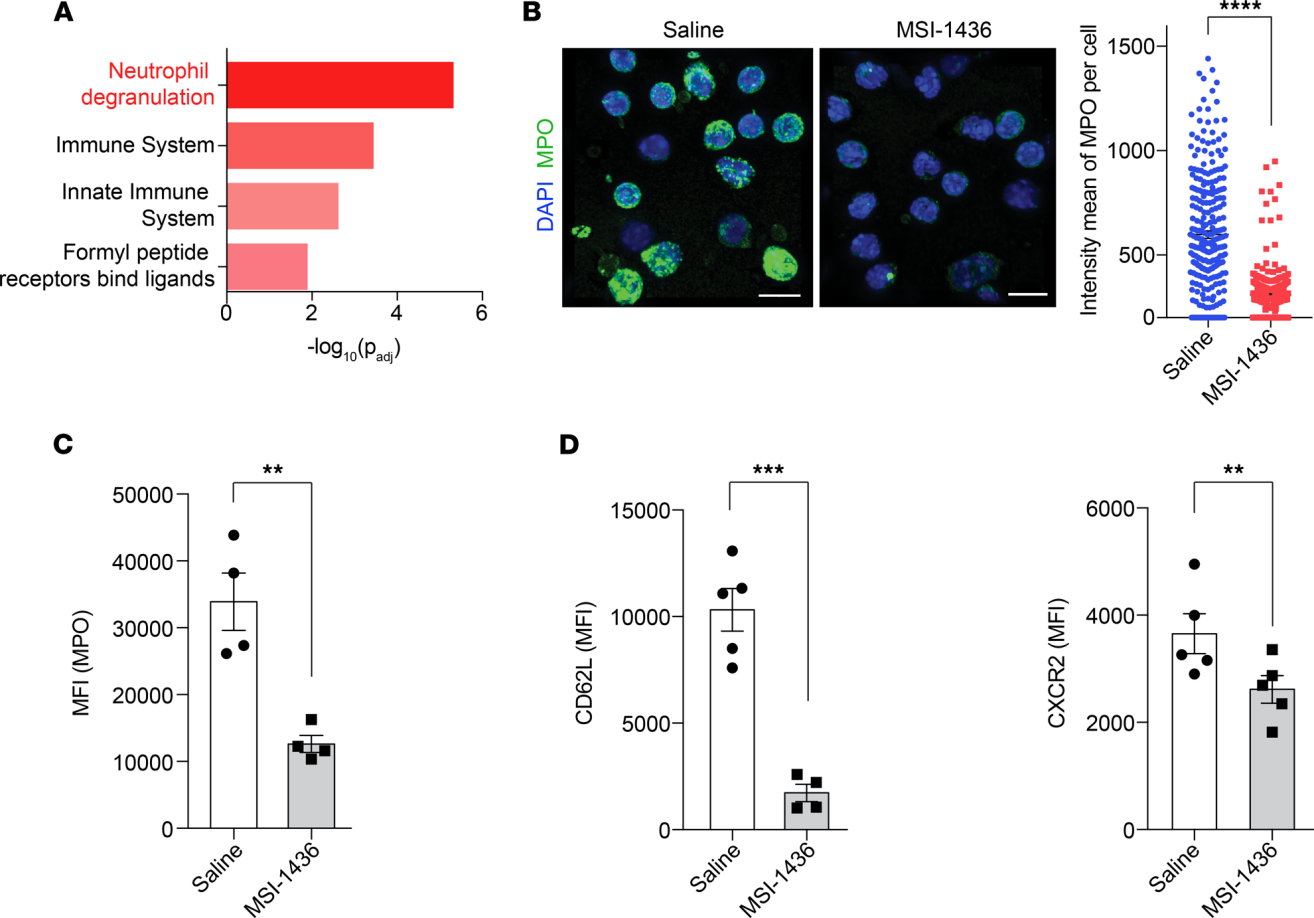
Treatment with MSI-1436 in vivo induced an aged-neutrophil phenotype. (**A**) Reactome pathway analysis performed using g:Profiler for genes upregulated upon MSI-1436 treatment. (**B**) Representative confocal immunofluorescence microscopy images and quantification of neutrophils isolated from peripheral blood 2.5 hours after injection of saline or MSI-1436, and stained with anti-MPO (green) and DAPI (blue). Scale bars: 10 μm, *n* = 4 mice per group. (**C**) Neutrophils stained for MPO-containing granules from mice treated with saline or MSI-1436 for 2.5 hours. MPO signals were quantified as mean fluorescence intensity (MFI) (*n* = 4). (**D**) Surface expression of CD62L and CXCR2 on neutrophils from peripheral blood collected 2.5 hours after saline or MSI-1436 treatment, quantified as MFI (*n* = 5). Data are presented as mean ± SEM. Statistical analysis for **B**–**D** by 2-tailed Student’s *t* test. **P < 0.01, ***P < 0.001, ****P < 0.0001.

Neutrophils display heterogeneity and plasticity, with the immune response determined more by the type of neutrophil subpopulation than by the number of neutrophils (34). Following administration of PTP1B inhibitors, TRALI-induced damage to the lung was minimal, despite increased neutrophil infiltration; to explore the mechanistic basis for this observation, we performed RNA-Seq to characterize phenotypic changes in neutrophils upon MSI-1436 treatment. We conducted Reactome pathway analysis on upregulated genes, which revealed that the most significantly altered pathway was neutrophil degranulation (Figure 3A and Supplemental Table 2).

The ability of neutrophils to clear pathogens is conferred primarily by 3 processes — degranulation, formation of NETs, and phagocytosis (35) — which are modulated during neutrophil aging (8, 9). Neutrophil granules contain antimicrobial and proteolytic proteins, which facilitate digestion of microorganisms in response to infection but have potential to cause harm to highly vascularized tissues, especially lungs, if not controlled appropriately. In the systemic circulation, neutrophils release granules in a controlled fashion, becoming less toxic and less able to cause tissue damage before they infiltrate the lungs (9). We harvested peripheral neutrophils 2.5 hours after treatment with either saline or MSI-1436 and stained for myeloperoxidase (MPO) as a marker of primary granules. We observed a pronounced decrease in immunofluorescence intensity of MPO in MSI-1436–treated neutrophils compared with saline-treated samples, suggesting the release of granules in vivo after treatment with the PTP1B inhibitor (Figure 3B). In addition, using flow cytometry, we demonstrate that the levels of MPO were decreased in the Ly6G^+^ neutrophil population isolated from MSI-1436– or DPM-1003–treated animals compared with saline-treated controls (Figure 3C and Supplemental Figure 4A).

There is a temporal heterogeneity, referred to neutrophil aging, in which fresh neutrophils are released from BM; they then undergo phenotypic changes to become aged neutrophils that are eventually eliminated from circulation (7). Compared with fresh neutrophils, intrinsically aged neutrophils display decreased granule contents and a reduced ability to form NETs, and their predominance in the circulation coincides with diminished risk for damage to the vascular system (8–10, 36). As a consequence, neutrophil aging is a physiological strategy to dampen the toxic nature of neutrophils before they infiltrate tissues, reducing their ability to cause tissue damage. Considering the degranulation phenotype we observed, we assessed the impact of treatment with PTP1B inhibitors on neutrophil aging by measuring the expression of surface markers for fresh neutrophils. Using flow cytometry, we demonstrate that, in MSI-1436–treated mice, the expression of CD62L and CXCR2 — 2 markers of fresh neutrophils — was downregulated (Figure 3D). Similarly, the markers of fresh neutrophils decreased upon DPM-1003 treatment (Supplemental Figure 4B). Collectively, these data suggest that PTP1B inhibitors promoted neutrophil aging in vivo and attenuated the neutrophil inflammatory response.

### Treatment with MSI-1436 suppressed formation of NETs ex vivo and in vivo.

**Figure 4:**
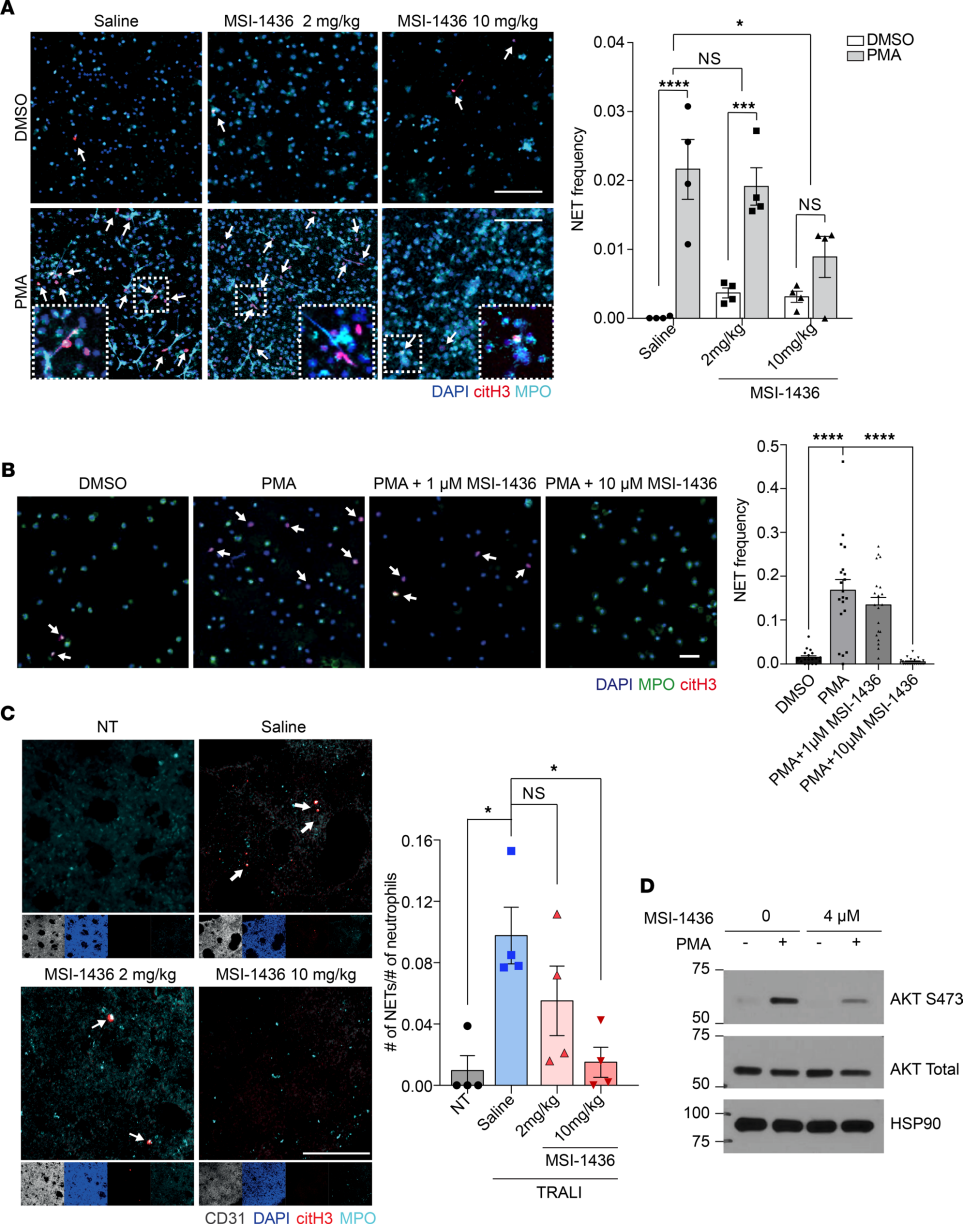
Treatment with MSI-1436 suppressed NET formation ex vivo and in vivo. (**A**) Representative immunofluorescence microscopy images and quantification, showing formation of NETs upon PMA treatment ex vivo. Arrows indicate NETs visualized by colocalization of DAPI (blue), citH3 (red), and MPO (cyan) staining. Higher magnifications of selected regions are shown in the lower squares. Scale bars: 100 μm, *n* = 4. The zoomed-in details for neutrophils treated with saline together with PMA (lower left panel), with 3 individual channels to show colocalization of DAPI, citH3, and MPO, is shown in Supplemental Figure 5A. (**B**) Representative confocal images and quantification, showing the NETosis frequency in response to PTP1B inhibitor and PMA stimulation. Scale bars: 50 μm, *n* = 4 mice per group with total 20 random fields for quantification. (**C**) Whole-mount staining of lung tissue from NT and TRALI mice administered saline, 2 mg/kg MSI-1436, or 10 mg/kg MSI-1436, with quantification of the frequencies of NETs. Arrows indicate NETs, visualized as in **A**. Scale bars: 100 μm, *n* = 4. (**D**) Immunoblot analyses showing AKT signaling changes using primary neutrophils isolated from BM. Representative immunoblot of 3 independent experiments. Data are presented as mean ± SEM. Statistical analysis for **A** was done by 1-way ANOVA with Dunnett’s multiple comparison test; by 1-way ANOVA with Sidak’s multiple-comparison test for **B**; and by 1-way ANOVA with Tukey’s multiple-comparison test for **C**. *P < 0.05, ***P < 0.001, ****P < 0.0001.

NETs are formed in a neutrophil cell death pathway, referred to as NETosis. In the classical form of NETosis, NADPH oxidase–induced ROS stimulate MPO to promote the translocation of neutrophil elastase (NE), a serine protease, to the nucleus and the decondensation of chromatin. Having observed that treatment with PTP1B inhibitors decreased MPO-containing primary granules, we examined whether release of NETs was also impaired. We harvested neutrophils 2.5 hours after MSI-1436 administration, and we stimulated them with phorbol 12-myristate 13-acetate (PMA), a protein kinase C (PKC) activator and trigger of ROS production, to induce NET formation ex vivo. NETs, which consist of DNA decorated with citrullinated-histone H3 (citH3) and granule proteins, were designated by colocalization of DNA, citH3, and MPO, using confocal microscopy. As shown in Figure 4A, the PMA-induced formation of NETs was blocked in neutrophils isolated from MSI-1436–treated animals, compared with PMA treatment of neutrophils from control, saline-treated animals.

We examined whether MSI-1436 suppressed NETosis through an intrinsic or extrinsic mechanism. The neutrophils were isolated from mouse circulating blood and incubated with either MSI-1436 or saline together with PMA. The PMA-induced NET formation was impaired by MSI-1436 treatment, indicating that this inhibitory effect was intrinsic to neutrophils (Figure 4B). Similarly, when we treated human primary neutrophils with MSI-1436 upon PMA stimulation, NET production was significantly reduced (Supplemental Figure 5B).

To determine whether MSI-1436 could also limit the production of NETs in the TRALI model, we treated mice with either saline or MSI-1436 and collected lungs 30 minutes after injection of anti–MHC-I antibody. We performed whole-mount immunostaining of the lung tissue for DAPI, citH3, and MPO. In the lungs of the TRALI mouse group treated with MSI-1436 at 10 mg/kg, the production of NETs was almost abolished (Figure 4C and Supplemental Videos 3 and 4). Furthermore, AKT signaling activated by NOX2-mediated ROS production is essential for NETosis (37); therefore, we tested the impact of PTP1B inhibitors on phosphorylation of Akt. We stimulated neutrophils isolated from BM with PMA or vehicle control, and we noted that both MSI-1436 and DPM-1003 suppressed the PMA-induced elevation of Akt phosphorylation (Figure 4D and Supplemental Figure 5C).

### The effect of PTP1B inhibitors on neutrophil aging was mediated via the CXCR4-CXCR2 signaling axis.

Our data indicate that treatment of TRALI mice with PTP1B inhibitors led to neutrophil aging, which, in turn, likely contributed to decreased lung damage compared with control animals. These observations phenocopied the genetic deletion of CXCR4 from neutrophils in mice (9), which led us to investigate whether there was a connection between inhibition of PTP1B and CXCR4 signaling.

**Figure 5:**
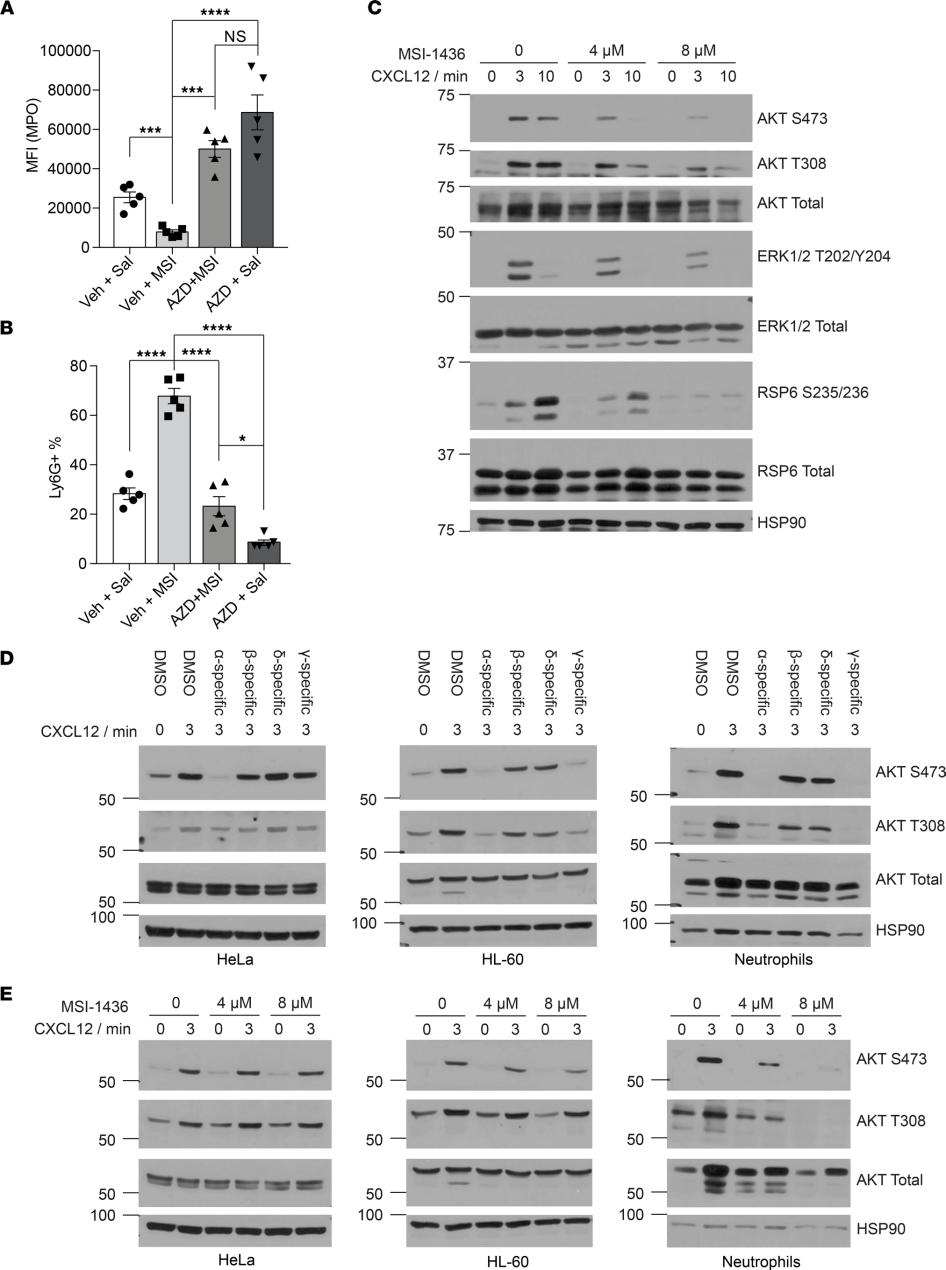
PTP1B inhibitors attenuated PI3Kγ-mediated CXCR4 signaling. (**A**) Quantitation of MPO from neutrophils isolated from mice treated with vehicle + saline, vehicle + MSI-1436, AZD5069 + MSI-1436, and AZD5069 + saline (*n* = 5 mice for each group). (**B**) Percentage of neutrophils, designated as Ly6G^+^ population, relative to total WBCs, from the indicated treatment groups (*n* = 5 mice for each group). (**C**) Immunoblot analyses showing the effect of MSI-1436 on CXCR4 signaling upon CXCL12 stimulation from primary neutrophils isolated from BM. Representative immunoblot of 4 independent experiments. (**D**) Immunoblot analyses showing the AKT signaling in response to PI3K isoform–specific inhibitors in HeLa, HL-60, and mouse neutrophils. Inhibitors used: α-specific (HS-173, 1 μM); β-specific (GSK2636771, 10 μM); δ-specific (Nemiralisib, 100 nM); γ-specific (Eganelisib, 200 nM); pretreated 1 hour before CXCL12 stimulation. Representative immunoblot of 3 independent experiments. (**E**) Immunoblot analyses showing the impact of pretreatment with MSI-1436 on AKT signaling in HeLa, HL-60, and mouse primary neutrophils. Representative immunoblot of 3 independent experiments. Data are presented as mean ± SEM. Statistical analysis for **A** and **B** by 1-way ANOVA with Tukey’s multiple-comparison test. *P < 0.05, ***P < 0.001, ****P < 0.0001.

The trafficking of neutrophils between BM and the circulation is controlled by the CXCR4/CXCR2 signaling axis (38). Stromal cells express a high level of CXCL12 (SDF-1), which interacts with CXCR4 and sequesters neutrophils in the BM (38). CXCL1 and CXCL2, which activate CXCR2 signaling, promote the egress of neutrophils into the blood stream (39). During neutrophil aging, the level of surface CXCR4 is upregulated, leading to clearance out of circulation and into the BM, among other tissues (40). To determine whether MSI-1436 acts through the CXCR4/CXCR2 axis, we tested whether AZD5069, a CXCR2 antagonist (41), could reverse the phenotype induced by MSI-1436. We administered AZD5069 orally, 2 hours prior to MSI-1436 injection, and collected blood samples to quantify the level of MPO using flow cytometry. As before, we found that the MPO fluorescence intensity decreased significantly in the MSI-1436–treated mice compared with vehicle-treated controls; however, pretreatment with AZD5069 was sufficient to prevent this reduction of the MPO signal in neutrophils of MSI-1436–treated mice (Figure 5A). In addition, although MSI-1436 treatment increased the Ly6G^+^ neutrophil population ~3-fold in serum, AZD5069 prevented this increase. Furthermore, treatment of mice with AZD5069 alone resulted in a lower percentage of Ly6G^+^ neutrophils compared with controls, which is consistent with the known function of CXCR2 to increase neutrophil release (Figure 5B). Altogether, these results demonstrate opposing effects of the CXCR2 antagonist AZD5069 and the PTP1B inhibitor MSI-1436, consistent with a stimulatory effect of PTP1B on the CXCR4/CXCR2 signaling axis.

### Treatment with PTP1B inhibitors impaired CXCR4 signaling.

Since CXCR4 inhibits the signaling output of CXCR2, and treatment with PTP1B inhibitors phenocopied ablation of CXCR4 from neutrophils in mice, we examined changes in signaling downstream of CXCR4. We purified neutrophils from BM, stimulated with the CXCR4 ligand CXCL12, and examined signaling changes by immunoblotting with appropriate antibodies. Class I phosphatidylinositol 3-kinases (PI3Ks) are activated downstream of GPCRs, including C-X-C chemokine receptors (CXCRs), and play an important role in neutrophil inflammatory response (42, 43). We observed that treatment with CXCL12 led to enhanced phosphorylation of AKT on Thr^308^ and Ser^473^, which are response markers for the abundance of PIP_3_ that is generated by PI3K; upon treatment with PTP1B inhibitors, we observed that phosphorylation of AKT, RPS6, and ERK1/2 were suppressed (Figure 5C and Supplemental Figure 6A). This result was unexpected because it has been well documented that PTP1B dephosphorylates the insulin receptor (IR) and thereby decreases AKT phosphorylation; consequently, activation of AKT might have been expected to accompany inhibition of PTP1B in this context (44, 45). Upon ligand binding to receptor tyrosine kinases or GPCRs, PI3Ks are recruited to the plasma membrane to activate AKT signaling. The catalytic subunit of PI3K, p110, consists of 4 isoforms, among which p110α, -β, and -δ are mainly activated by receptor tyrosine kinases, whereas p110γ is activated by G protein βγ dimer (46). Therefore, since p110γ is preferentially expressed in leukocytes, we hypothesized that PTP1B inhibitors may, instead, exert suppressive effects on p110γ-dependent AKT signaling. We used PI3K isoform selective inhibitors to study the contribution of different p110 isoforms in regulating AKT downstream of CXCR4. We used the HL-60 promyelocytic cell line and mouse primary neutrophils to examine cells from the myeloid lineage, and we used HeLa (PI3K^WT^ and PTEN^WT^) as a nonleukocyte control. After CXCL12 stimulation of HeLa cells, the phosphorylation of AKT was suppressed by the PI3Kα inhibitor, HS-173 (47), whereas both HS-173 and the PI3Kγ inhibitor Eganelisib (48) inhibited CXCL12-dependent AKT phosphorylation in neutrophils and HL-60 cells. In contrast, inhibition of PI3Kβ, using GSK2636771 (49), and inhibition of PI3Kδ, using Nemiralisib (50), exerted much less effect (Figure 5D) in all of the cells tested. To examine whether the attenuation of CXCL12-dependent AKT phosphorylation in response to PTP1B inhibitors was mediated by PI3Kγ, we pretreated cells with MSI-1436 and DPM-1003. Both PTP1B inhibitors exerted minimal effect on CXCL12-induced AKT phosphorylation in HeLa cells. In contrast, they suppressed AKT signaling in response to CXCL12 in HL-60 cells and neutrophils, suggesting that PTP1B inhibitors impaired PI3Kγ-mediated AKT signaling downstream of CXCR4 (Figure 5E and Supplemental Figure 6B).

To assess further the specificity of PTP1B inhibitors on CXCR4 signaling, we evaluated their effects on 2 additional GPCRs. Upon stimulation of CXCR2 with CXCL2, downstream signaling was activated, and PTP1B inhibitors attenuated AKT phosphorylation (Supplemental Figure 6, C and D). In contrast, treatment with PTP1B inhibitors enhanced *N*-formyl-methionine-leucine-phenylalanine–induced (fMLP-induced) AKT phosphorylation (Supplemental Figure 6, E and F), suggesting that the suppression of GPCR signaling was agonist and receptor dependent.

### mTOR inhibitor improved survival in the TRALI model and induced an aged-neutrophil phenotype.

**Figure 6:**
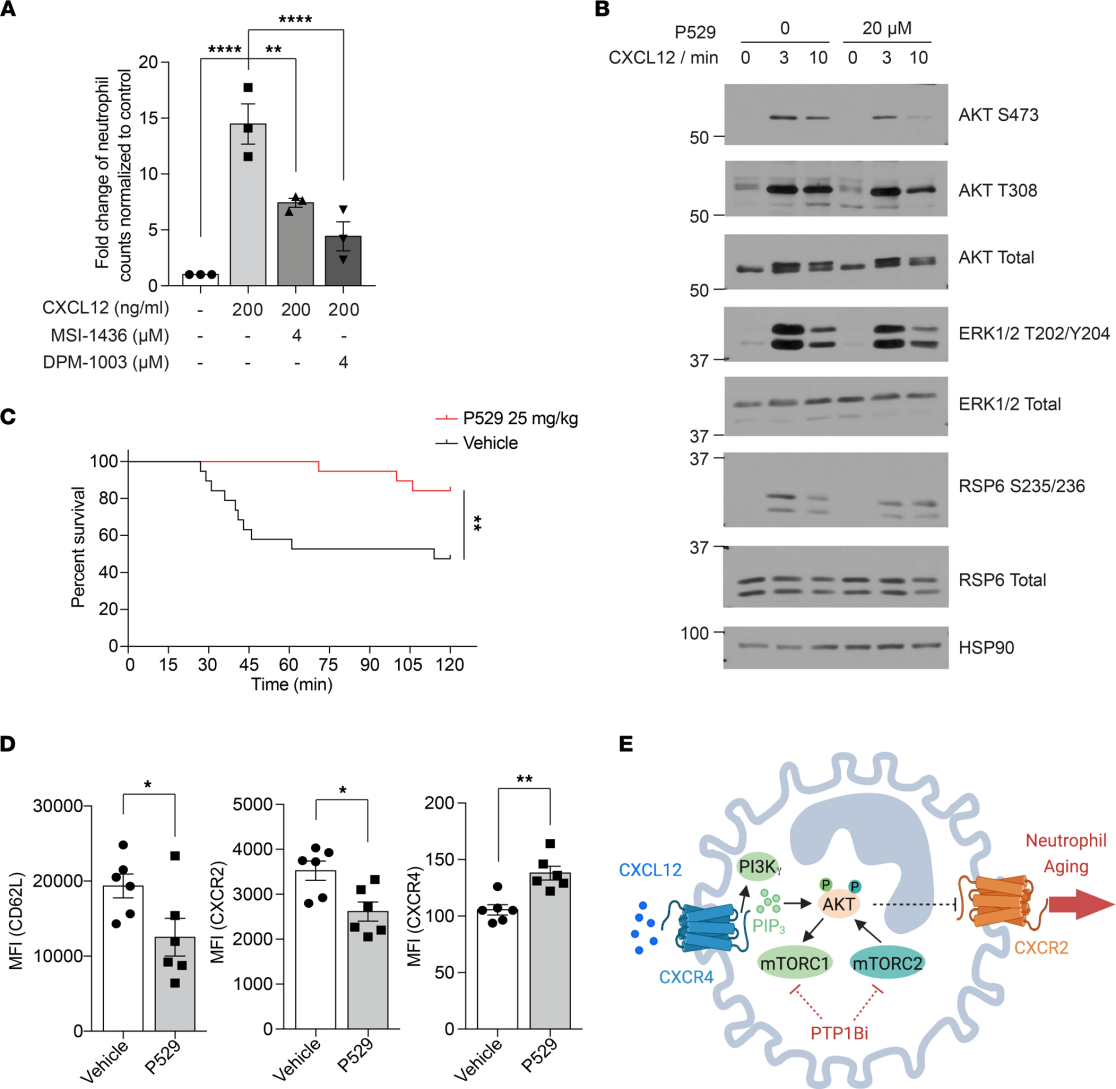
The effect of mTOR inhibitor on the survival of TRALI model and induction of aged-neutrophil phenotype. (**A**) Neutrophil migration toward CXCL12 examined using Transwell assays (*n* = 3 independent biological repeats). (**B**) Immunoblot analyses showing the effect of P529 on mTOR signaling upon CXCL12 stimulation from primary neutrophils isolated from BM. Representative immunoblot of 3 independent experiments. (**C**) Survival curve of TRALI mice treated with vehicle or 25 mg/kg P529 (*n* = 19 mice, 2 independent biological repeats). (**D**) Surface expression of CD62L, CXCR2, and CXCR4 on neutrophils from peripheral blood collected 2.5 hours after vehicle or P529 treatment, quantified as MFI (*n* = 6 mice for each group). (**E**) Schematic representation of the proposed mechanism, created with BioRender.com. Data are presented as mean ± SEM. Statistical analysis for **C** by log-rank (Mantel-Cox) test and by 2-tailed Student’s *t* test for **D**; *P < 0.05, **P < 0.01, ****P < 0.0001.

In the studies above, we observed a pronounced suppression of AKT phosphorylation at residue Ser^473^ by PTP1B inhibitors; this phosphorylation is mediated by mammalian target of rapamycin complex 2 (mTORC2) (51). AKT that has been phosphorylated at Thr^308^ indirectly activates mTORC1, which activates S6 kinase 1 to phosphorylate RPS6 (52). The decreased phosphorylation of AKT, RPS6, and ERK1/2 suggested PTP1B inhibitors suppressed mTOR-mediated CXCR4 signaling. It has been reported that mTORC2 plays a role in chemoattractant-stimulated neutrophil chemotaxis by regulating myosin II in a RhoA-dependent manner (53). To evaluate whether inhibition of PTP1B also affected migration of neutrophils toward chemotactic cues, we used a Transwell assay to assess their response to CXCL12. Compared with the control, pretreatment of either MSI-1436 or DPM-1003 significantly impaired the migration of neutrophils (Figure 6A). Consistent with the effect of PTP1B inhibitors promoting fMLP-dependent AKT signaling, both inhibitors enhanced the migration of neutrophil toward fMLP (Supplemental Figure 7, A and B).

Since PTP1B inhibitors promoted an aged-neutrophil phenotype through suppression of mTOR, we examined whether mTOR inhibitors act similarly to PTP1B inhibitors. We observed that the CXCL12-stimulated phosphorylation of AKT Ser^473^ was attenuated following treatment with Palomid 529 (P529) (Figure 6B), an inhibitor of mTORC1 and mTORC2 complexes that has been reported to decrease phosphorylation of AKT on Ser^473^ (54). Furthermore, treatment with P529 two hours before TRALI induction improved overall survival at 2 hours to more than 80%, compared with less than 50% in the vehicle-treated control group (Figure 6C). In the LPS-induced sepsis model, pretreatment of P529 two hours prior to LPS (15 mg/kg) challenge significantly prolonged the survival time compared with the vehicle control mice (Supplemental Figure 7C). Similar to PTP1B inhibitor treatment, P529 treatment promoted an aged-neutrophil phenotype, including downregulation of fresh neutrophil markers CD62L and CXCR2, and upregulation of the aged-neutrophil marker CXCR4 (Figure 6D). These findings highlight inhibition of PI3Kγ/AKT/mTOR-mediated CXCR4 signaling as a component of the mechanism by which PTP1B inhibitors protected against lung injury in the TRALI model and improved survival in LPS-induced sepsis model (Figure 6E).

## Discussion

Neutrophils, which are the most abundant WBC type, play an important role in the innate immune response, providing protection from invading pathogens (5). These beneficial antmicrobial functions, which include phagocytosis, degranulation, and NET formation, have to be balanced with potentially deleterious inflammatory effects. This balance is achieved, in part, through a neutrophil aging process that follows a circadian rhythm and contributes to the homeostasis of neutrophil number and phenotypic status (7). Neutrophils, which are produced from hematopoietic stem cells in the BM, differentiate into a mature form that is enriched in the granules and secretory vesicles that underlie the microbicidal function (55). Upon their controlled release into the bloodstream, the neutrophils circulate throughout the body and distribute to the sites of infection or inflammation in various tissues. Finally, as they spend time in circulation, they suffer a process of aging that renders them prone to be cleared out of circulation and into the tissues, where they are eliminated by macrophages and DCs (56). It has now been established that neutrophils undergo morphological changes, from when they leave the BM as fresh neutrophils until they age and are cleared from circulation (7). In this current study, we demonstrate that a single dose of either of 2 distinct allosteric inhibitors of PTP1B induced a phenotype that exhibited features of neutrophil aging. This coincided with attenuation of lung injury and decreased mortality in a murine, neutrophil-dependent TRALI model of ARDS and the LPS-induced endotoxemia model of sepsis. Furthermore, PTP1B inhibitor MSI-1436 also improved survival in the CLP-induced model of sepsis.

Both MSI-1436 and DPM-1003 are allosteric inhibitors that primarily target the noncatalytic, disordered segment in the C-terminus of PTP1B. This segment is a unique portion of the PTP1B protein that is unrelated to TC-PTP (T cell protein tyrosine phosphatase, encoded by *PTPN2*), its closest relative, or to any other member of the PTP family (19). Consequently, we expect that such inhibitors have the potential to be highly specific for PTP1B over other members of the PTP family. In our initial study of the impact of MSI-1436 on PTP1B in models of breast cancer, we reported a double-mutant, PTP1B-L192A/S372P, in which catalytic function was preserved but inhibition by MSI-1436 was abrogated (19). Following expression of this mutant in tumor cells, inhibition of cell migration and growth of tumor xenografts by MSI-1436 was markedly attenuated, consistent with PTP1B being a major target through which the compound exerted its effects. In this current study, we observed improvement of survival in the TRALI model at 2 mg/kg MSI-1436 and 5 mg/kg DPM-1003; the plasma concentrations of the compounds at these doses are also consistent with their effects being exerted through inhibition of PTP1B. Furthermore, the fact that both inhibitors produce consistent effects in this study also highlights the importance of PTP1B as a target. Nevertheless, this does not exclude a contribution of additional effects on other targets. At this time, due to technical challenges posed by differences in genetic backgrounds of the various mouse models, it is not possible to incorporate complementary genetic studies in PTP1B-ablated animals.

The process of neutrophil aging features a cell-intrinsic signaling module, in which chemokine receptors CXCR2 and CXCR4 functionally oppose one another. CXCR2 promotes mobilization of neutrophils into the blood stream, whereas CXCR4 retains neutrophils in the BM, with CXCR4 playing a dominant role over CXCR2 (38). As neutrophils are released into the blood, they undergo a process of aging whereby they downregulate the expression of CXCR2, upregulate the expression of CXCR4, and undergo microanatomical and proteomic changes that ultimately promote their homeostatic clearance out of circulation and into tissues, such as BM, where the level of the chemokine ligand CXCL12 is constitutively high (57). Aged neutrophils, therefore, are prone to be cleared out of circulation and have reduced ability to respond to inflammatory stimuli. In the context of neutrophil aging, the signaling module is driven by BMAL1, a transcription factor that regulates the circadian clock (58). BMAL1 controls the expression of CXCL2, a chemokine ligand of CXCR2, to promote neutrophil aging; in contrast, CXCR4 impairs the aging process (8). In fact, deletion of *Cxcr4* from neutrophils promotes the acquisition of an aged phenotype. It is interesting to note that treatment of mice with inhibitors of PTP1B phenocopies the neutrophil loss of CXCR4 — including neutrophilia, progressive loss of granule content, downregulation of fresh neutrophil markers CD62L and CXCR2, and decreased formation of NETs — upon inflammatory challenge in the lung. This suggests that one mechanism by which our inhibitors of PTP1B exert their effects is through suppression of the inhibitor of aging, CXCR4.

CXCR4 is a GPCR that can activate diverse downstream signaling pathways (59). Among them, PI3K plays an important role in regulating neutrophil migration, ROS generation, and the respiratory burst (42). In leukocytes, PI3Kγ is the preferentially expressed isoform, and its activity is regulated by G protein βγ heterodimers (46). Mice deficient in the p110γ PI3K catalytic subunit showed a higher number of neutrophils in the circulation, impaired neutrophil migration, and ROS generation — phenotypes similar to the mouse model of *Akt2* deletion (42, 60, 61). In this study, following treatment with our PTP1B inhibitors, we observed neutrophilia, reduced phosphorylation of AKT, and attenuated neutrophil migration toward CXCL12. These observations suggest that PTP1B inhibitors function to inhibit PI3Kγ/AKT signaling downstream of CXCR4. In accordance with the reduction in NET formation following suppression of AKT (37), we show that MSI-1436 is a potent inhibitor of PMA-mediated NETs in vitro and NET formation in the lung tissues during TRALI.

The phosphorylation of AKT on Thr^308^ is mediated by 3-phosphoinositide-dependent protein kinase-1 (PDK-1), whereas phosphorylation of Ser^473^ is mediated by mTORC2 (43, 62). It has been shown that mTORC2 controls neutrophil chemotaxis by cAMP/RhoA-dependent phosphorylation of Myo-II, which regulates tail retraction and adhesion (53, 63). In our study, we demonstrate an impaired chemotactic response to the CXCR4 ligand CXCL12 upon PTP1B inhibitor treatment. Furthermore, we observed an aged-neutrophil phenotype upon pharmacological inhibition of mTOR with P529. Our observation that P529 partially rescued mortality in the TRALI model and prolonged survival in LPS-induced sepsis model suggests that inhibitors of PTP1B function, at least in part, through suppressing AKT/mTOR-mediated CXCR4 signaling, to promote neutrophil aging. Compared with fresh neutrophils, intrinsically aged neutrophils have reduced propensity to migrate into inflamed tissues, less granule content, and lower capacity to produce NETs (8, 9). As a consequence, neutrophil aging is a physiological strategy to dampen the cytotoxic nature of neutrophils before they infiltrate into tissues and to prevent tissue damage. Nevertheless, there are also reports suggesting that neutrophil aging mediated by extrinsic stimuli, such as microbiota, favor a proinflammatory phenotype (64, 65). The mechanism by which such extrinsic signals would be coordinated with the intrinsic program requires further investigation.

It is interesting to note that, in the presence of multiple stimuli, the chemotactic response of neutrophils is processed in a hierarchical manner, prioritizing “end target” (such as fMLP) over “intermediary” (such as CXCL2, CXCL12) chemoattractants to determine the direction of migration (66). This preference is consistent with differences in the nature of the signaling events downstream of the cognate GPCRs and context-dependent effects of phosphatases, such as PTP1B, on signaling outcome (67). In our study, we highlight specificity in the effects of PTP1B inhibitors in suppressing signaling in response to CXCL2 and CXCL12, but in enhancing fMLP-induced signaling. In response to fMLP, activation of p38 MAPK promotes neutrophil migration. PTP1B dephosphorylates p38 MAPK directly (13), which likely explains why PTP1B inhibition enhanced fMLP-mediated signaling. In contrast, intermediary chemokines primarily function through PI3K (68), consistent with a different point of action of PTP1B.

It is important to emphasize that the old ideas that phosphatases are merely housekeeping enzymes that are there simply to dephosphorylate, and antagonize the activity of, protein kinases are no longer borne out by the data (67, 69, 70). It is now well established that phosphatases are, themselves, critical regulators of signaling in their own right, with the potential to function both negatively and positively, depending upon the context (67). This has been established already for PTP1B. PTP1B can serve to dephosphorylate and inactivate the IR and the leptin receptor–associated kinase JAK2; this is what laid the foundation for excitement about PTP1B as a therapeutic target for diabetes and obesity (71, 72). Considering this precedent, it would have been expected that inhibition of PTP1B would promote PI3Kα-mediated AKT, rather than the attenuation of AKT signaling we observed in neutrophils. Nevertheless, it is already established that, in different contexts, PTP1B serves as a positive regulator of signaling — for example, downstream of the HER2 oncoprotein tyrosine kinase (73, 74). In such cases, inhibition of PTP1B would be expected to suppress signaling. In the context of GPCR, the Gβγ heterodimer promotes the activation of PI3Kγ. In fact, we observed that MSI-1436 and DPM-1003 exerted minimal effect on CXCL12-induced AKT phosphorylation in HeLa cells. In contrast, they suppressed AKT signaling in response to CXCL12 in HL-60 cells and neutrophils, suggesting that PTP1B inhibitors impaired PI3Kγ-mediated AKT signaling downstream of CXCR4. Our observations, which provide a further illustration of specificity in PTP1B function in a biological context, are consistent with previous studies reporting that PTP1B inhibition attenuates mTOR signaling through activating AMPK, which suppressed mTOR activity in mast cells, pancreatic cancer cells, and hepatocytes (75–77). In addition, the phosphorylation of mTOR at Ser^2448^ is decreased in BM-derived macrophages isolated from myeloid PTP1B-depleted mice (78). The identification of target substrates of any phosphatase is a formidable undertaking, and at this time, the identity of the direct substrates of PTP1B that underlie the control of PI3Kγ/AKT/mTOR signaling is unclear and requires further study.

It is important to note that PTP1B has been implicated in various immune responses, including attenuation of CD40 and BAFF-R pathways in B cells (13), negative regulation of the JAK/STAT5 pathway in T cells (79), and modulation of macrophage activities (14–16). Neutrophils are important pathological drivers in ARDS and many other inflammatory diseases, such as multiple sclerosis, psoriasis, and chronic obstructive pulmonary disease (80–82). Thus, manipulation of neutrophil aging to dampen the neutrophil activity may be an attractive antiinflammatory therapeutic approach. In this study, we demonstrate that PTP1B inhibitors prevent lung injury in the TRALI model and improve survival in CLP- and LPS-induced sepsis models via a mechanism to promote neutrophil aging. MSI-1436, the prototypic allosteric PTP1B inhibitor, was well tolerated by patients enrolled in clinical trials for obesity and type 2 diabetes (T2D) (83) and breast cancer. This, coupled with the beneficial effect in ameliorating ALI, highlights PTP1B inhibitors as potential therapeutics for treating neutrophil-mediated lung damage, an indication that has risen to prominence recently due to the impact of lung injury in patients with advanced COVID-19 disease.

## Methods

Supplemental Methods are available online with this article.

### Statistics.

Statistical analyses were performed with Prism software (GraphPad Software). All data are presented as mean ± SEM. Comparison between 2 groups were analyzed by unpaired 2-tailed *t* test. Comparison for more than two groups were analyzed using one-way ANOVA followed by indicated post-hoc test. Statistical significance of Kaplan-Meier curves was determined by Log-rank (Mantel-Cox) test.

### Study approval.

All the mouse studies were performed in accordance with procedures approved by the IACUC at CSHL and NIH Guide for Care and Use of Laboratory Animals. Whole blood from healthy volunteers was collected with informed consent and approved by the IRB of CSHL (IRB-13-025).

## Author contributions

DS, JMA, SKL, TJ, ME, and NKT designed experiments. DS, JMA, CF, LNC, XYH, and JRM performed experiments. JEW performed histological analyses. DS, JMA, XYH, JRM, and NKT analyzed the data. DS and NKT wrote the manuscript, and all authors contributed to editing. NKT conceptualized and supervised the study.

## Supplementary Material

Supplemental data

Supplemental video 1

Supplemental video 2

Supplemental video 3

Supplemental video 4

## Version 1 07/22/2022

Electronic publication
