# Supplementary material for: PTP1B inhibitors protect against acute lung injury and regulate CXCR4 signaling in neutrophils
Source: JCI Insight. 2022 Jul 22;7(14):e158199. doi: 10.1172/jci.insight.158199 (PMC9431713; doi:10.1172/jci.insight.158199)
Supplement: Supplemental data [file jciinsight-7-158199-s123.pdf]

Supplemental material for

PTP1B inhibitors protect against acute lung injury and regulate CXCR4 signaling in neutrophils.

Dongyan Song[1,2], Jose M. Adrover[1], Christy Felice[1], Lisa N. Christensen[1], Xue-Yan He[1], Joseph R. Merrill[1], J. Erby Wilkinson[3], Tobias Janowitz[1], Scott K. Lyons[1], Mikala Egeblad[1] and Nicholas K. Tonks[1]*

Affiliations:

[1]Cold Spring Harbor Laboratory, Cold Spring Harbor, New York 11724, USA;

[2]Molecular and Cellular Biology Program, Stony Brook University, Stony Brook, New York 11794, USA;

[3]Unit for Laboratory Animal Medicine, Department of Pathology, University of Michigan, Ann Arbor, Michigan 48109, USA.

*To whom correspondence should be addressed:

Email: tonks@cshl.edu

Materials and Methods

Mice. Male Balb/c mice (7-14-week-old) were obtained from Charles River and male C57BL/6J mice (7-10-week-old) were purchased from The Jackson Laboratory. All the mice were housed in the animal facilities of Cold Spring Harbor Laboratory. All experimental protocols were reviewed and approved by the Cold Spring Harbor Laboratory Institutional Animal Care and Use Committee (IACUC) and were conducted in accordance with the NIH's Guide for the Care and Use of Laboratory Animals. Mice were housed five per cage and maintained on a 12h light/dark cycle and an ambient temperature of 25°C, with sterile food and water, in conventional space. All mice were acclimatized to the animal facility for a minimum of 7 days prior to enrollment in experiments.

Cell culture and cell lines. HL-60 and HeLa cell lines were obtained from ATCC. HL-60 cells were cultured in Iscove's Modified Dulbecco's Medium (IMDM) supplemented with 20% FBS and 100 U/ml penicillin/streptomycin. HeLa cells were cultured in Dulbecco's Modified Eagle Medium (DMEM) supplemented with 10% FBS and 100 U/ml penicillin/streptomycin. Cells were grown in a 37 °C humidified incubator with 5% CO_2.

TRALI induction. Male Balb/c mice (7-9-week-old) were given an intraperitoneal injection with 0.15 mg/kg lipopolysaccharide (LPS) in 100 μl physiological saline (0.9%). Twenty-six hours later, mice received an intravenous injection through retro-orbital venous sinus of 100 μl 1.5 mg/kg anti-mouse MHC Class I (BioXcell, clone 34-1-2s). Two hours before TRALI induction, the mice were treated either with saline or different doses of MSI-1436 or DPM-1003, intraperitoneally. Due to the circadian oscillation of neutrophils, anti-MHC-I was administered at ZT13 (7 p.m.) Mice were observed for up to 2hrs during the acute phase of TRALI. Mice were euthanized when they appeared moribund by physical inspection as evidenced by change in mobility (endpoint of the experiment). Time to endpoint was used for statistical analysis of overall survival.

CLP-induced sepsis. Male C57BL/6J mice (8-10-week-old) were administered a subcutaneous injection in the flank of analgesic (72-hour sustained release Buprenorphine (1 mg/kg)) 15 min before surgery. Then, mice were anesthetized with isoflurane, and surgery was performed under aseptic survival conditions. A small midline incision was made and the cecum was exteriorized and ligated (2 cm) using non-absorbable 3-0 suture. A 23-gauge needle was then used to puncture one hole in the middle of the ligated segment, and a small amount of feces was extruded to ensure constant drainage of puncture. The ligated and punctured cecum was repositioned inside of abdominal cavity without the feces touching the incision to avoid infection of the surgical wound. The peritoneum was closed with surgical absorbable Vicryl sutures, and the skin was closed using sterile 7 mm wound clips. Survival was monitored for 10 days, and mice were euthanized once they became moribund and a humane endpoint was reached.

LPS-induced sepsis. Male C57BL/6J mice (7-9-week-old) were given LPS (*E. coli* O111:B4) at the indicated concentrations through intraperitoneal injection. Two hours before LPS challenge, 10 mg/kg MSI-1436 or saline were administered intraperitoneally. Mice were observed for up to 5 days for survival, and mice were euthanized once they became moribund.

Histology. Two lung fixation methods were used in this study. For both techniques, animals were euthanized with CO_2 immediately prior to procedure. The first technique required exposing the trachea and lung, followed by making an incision in the trachea to allow insertion of a 20-gauge catheter (Exel Safelet catheter). The catheter and trachea were secured with sutures and the lungs were slowly inflated with approximately 1ml of 4% paraformaldehyde (PFA). The trachea was then tied to prevent deflation and the lungs were dissected and immersed in 4% PFA at room temperature for 24 hours to ensure thorough fixation. For the second technique, animals were first transcardially perfused with 30ml of physiological saline solution (0.9%), to flush out blood, using a 25-27GA needle. The lungs were

then inflated by repositioning the needle from the left ventricle of the heart into the right ventricle and perfusing with an additional 3-5ml of saline. The lungs were dissected and drop fixed in 4% PFA at room temperature for 24 hours. After dehydration, the fixed lungs were embedded in paraffin and 5 μm sections were cut coronally, to represent all the lobes, and mounted on slides. Tissue sections were stained with hematoxyline and eosin (H&E), and scanned using Aperio ScanScope CS (Leica Biosystems).

All H&E staining slides were read blindly and scored for lung damage. H&E-stained sections were scored according to severity, distribution, amount and content of edema, alveolar damage, hyaline membranes and vessel damage. Severity was scored 0-4 based on the least to most affected lung in the study. Distribution was scored 0-4 based on the percentage of the lung involved (focal, multifocal, locally extensive, and diffuse). Edema scores were 0-4 based on the distribution, severity, and intensity of the edematous proteinaceous exudate in the alveolar space. Alveolar damage was scored 0-4 according to the degree of loss of alveolar wall integrity and alveolar pneumonocyte reaction (type II hyperplasia and sloughed cells in alveoli). Hyaline membranes were scored according to number and extent of membrane formation. Vessel damage was scored 0-4 according to the degree of endothelial damage. The scores of each lesion were added to give a final overall score.

Quantification of bronchoalveolar lavage fluid (BALF) protein. The BALF was collected from NT, saline or 10 mg/kg MSI-1436 treated TRALI mice. Mice were euthanized in a CO_2 chamber. For the TRALI group, BALF extraction was performed 45 minutes after MHC-I antibody injection, or postmortem if mice died earlier than 45 minutes. Each mouse was positioned front side up, with the trachea exposed. A silk thread (Perma-Hand silk, 3-0, Ethicon) was placed under the trachea. After the trachea were hemisected transversally, a 20G catheter (Exel Safelet catheter 20G 1'', Exelint) was inserted into the trachea, and tied firmly with silk thread. PBS (1ml) was injected into the lungs, and slowly recovered after ~ 1

minute. A total of 600 -700 μl BALF was recovered from each mouse, and kept on ice. BALF was centrifuged (300xg, 10 min, 4°C), and the protein concentrations in the supernatants were determined using Pierce BCA Protein Assay Kit (Thermo Fisher Scientific) according to the manufacturer's instructions.

CT scan for lung edema measurement. Balb/c mice were anesthetized with 120 mg/kg ketamine and 8 mg/kg xylazine. After immobilization, mice were positioned prone on the imaging cradle of either a Mediso nanoScan PET/CT or SPECT/CT system (Mediso), and secured with tape and gauze to prevent movement. The scanner's field of view was set using a 2D scout scan to cover the lungs and airways, and images were acquired using the following x-ray settings: beam energy of 50 kVp, exposure of 186 μAs, in an axial scan with 720 projections. After acquisition of each baseline CT scan, the mouse was injected with 1.5 mg/kg antibody against MHC-I retro-orbitally, immediately followed by sequential scans that were acquired at 5-minute intervals for 45 minutes, or until endpoint. To minimize the effect of circadian rhythm on edema accumulation, TRALI was induced for one saline- and one MSI-1436-treated mouse at similar times (6 p.m. – 10 p.m.), and placed alternately on either CT scanner.

Images were reconstructed using a back-projection algorithm with a Butterworth filter to an isotropic voxel size of 138 μm in Nucline software v3.0 (Mediso). Pre-processing and analyses were performed in VivoQuant v4.0 (Invicro). Briefly, a 3D region of interest (ROI) covering the entire lung volume was semi-manually contoured on the baseline scan and the first follow-up scan after antibody injection, with the latter ROI being used on all subsequent scans as the mouse was not moved. Total lung volume and mean image intensity in Hounsfield units (HU) were calculated from the ROI at each imaging timepoint. The viable lung volume was defined as the volume within the total lung ROI below a threshold of 0 HU, and percent viable lung volume was calculated by dividing this value by the total lung volume at each timepoint.

Blood count. Blood was collected by cardiac puncture into a syringe freshly coated with 0.5 M EDTA, then transferred into EDTA-coated tubes (Microvette 500, Sarstedt). We ensured that blood used for functional analysis in this study was clot free, in order to avoid neutrophil activation. Blood (50 μl) was analyzed for differential counts using a ProCyte Dx Hematology Analyzer (Idexx Laboratories).

Lung immune cell infiltration analysis. Lung tissues were harvested from control and TRALI mice 30 minutes post MHC-I antibody challenge. Lungs were rinsed in cold PBS and mechanically dissociated into small pieces, and further enzymatically digested for 30 min at 37 °C in 5 ml of RPMI with 2% FCS and containing Dispase (2.5 U/ml, #07913, Stem Cell), Collagenase D (0.1 mg/ml, #11088866001, Sigma), DNase I (25 U/ml, #04536282001, Sigma), and Liberase DL (0.2 mg/ml, #05466202001, Sigma). The suspensions were then passed through a 70 μm cell strainer (#352340, BD Falcon), and centrifuged at 1,500 rpm for 5 minutes at 4 °C. After removing the red blood cells by incubating with 5 ml of ammonium-chloride-potassium (ACK) buffer for 3 min on ice, the suspensions were then centrifuged (1,500 rpm, 5 min) and resuspended in FACS buffer (1% FCS and 0.02% sodium azide in PBS). The single-cell suspensions were finally collected by passing through a 40 μm cell strainer. For flow cytometry analysis, lung single-cell suspensions (1,000,000 cells) from each sample were fixed in 2% of PFA for 10 min on ice. After washing with FACS buffer, the cells were blocked with Fc Receptor Blocker (Innovex Biosciences, Richmond, CA) for 15 min at 4 °C, followed by incubating with conjugated antibodies (listed in the table below) for 30 min at 4 °C. Finally, the cells were washed once in FACS buffer and resuspended in 500 μl of FACS buffer and further analyzed by BD LSRFortessa DUAL flow cytometer (BD Biosciences). Data were analyzed by FlowJo software (V10.6.2). Immune cell subsets and corresponding markers: CD8+ T cells (CD45+CD3+CD8+); CD4+ T cells (CD45+CD3+CD4+); γδ T cells (CD45+CD3+gdTCR); NKT cells (CD45+CD3+CD335+); B cells (CD45+CD3-CD19+); NK cells (CD45+CD3-CD335+); Neutrophils

(CD45+CD11b+Ly6G+Ly6C+); Macrophages (CD45+CD11b+Ly6G-F4/80+); and DC cells (CD45+CD11b+Ly6G-F4/80-CD11c+).

Antibody	Clone	Species	Manufacturer
CD45-BV785	30-F11	Mouse	BioLegend
CD3-Alexa Fluor 488	17A2	Mouse	BioLegend
CD4-Alexa Fluor 647	GK1.5	Mouse	BioLegend
CD8-PE	53-6.7	Mouse	BioLegend
CD19-PE/Cy5	6D5	Mouse	BioLegend
CD45-APC	30-F11	Mouse	BD Biosciences
CD335(NKp46)-eFluor 450	29A1.4	Mouse	Invitrogen
TCR gamma/delta-PE	eBioGL3	Mouse	Invitrogen
CD11b-PE	M1/70	Mouse/Human	BD Biosciences
CD11c-PE/Cy7	N418	Mouse	BioLegend
Ly6C-APC	HK1.4	Mouse	BioLegend
Ly6G-FITC	1A8	Mouse	BioLegend
F4/80-PerCP/Cy5.5	BM8	Mouse	BioLegend

Multiplex cytokine array. The cytokine arrays were performed with serum and lung tissue homogenates from NT (no treatment) and TRALI mice 30 min after MHC-I antibody injection. For the serum samples, blood was collected by cardiac puncture, then allowed to clot for 30min at room temperature. The serum was isolated by centrifuging at 1,500 x g for 15 minutes in a refrigerated centrifuge. For lung tissue homogenates, lungs were harvested and weighed, homogenized using a Precellys Evolution tissue homogenizer (Bertin Instruments) at 6,800 rpm, 0°C. The samples were homogenized for three cycles, 20 s per cycle, and 30 s pause after each cycle. Samples were analyzed with the Proteome Profiler mouse cytokine array kit, panel A (R&D systems, ARY006) according to the manufacturer's instructions.

Enzyme-linked immunosorbent assay (ELISA). ELISA was performed with serum, plasma or lung homogenates to measure the levels of CXCL1 (R&D systems, DY453-05) and CXCL2 (R&D systems, DY452-05) following manufacturer's instructions.

Isolation of bone marrow (BM) neutrophils. BM neutrophils were isolated by density gradient centrifugation, as previously described (1). Briefly, BM was flushed from tibias and femurs using HBSS. The cell pellet was resuspended in ACK buffer and passed through 100 μm cell strainer (Falcon). Different concentrations of Percoll (Cytiva, 17089102) were prepared according to previous publication (2). Neutrophils were enriched using gradient centrifugation at 1,300 x g for 20 min, and collected from the band at the interface between the 81 and 62% Percoll layers. Cells were washed with HBSS, and resuspended in RPMI at the desired concentration.

RNA isolation and RNA-seq library preparation. Neutrophils were isolated from bone marrow (BM), and treated with either saline or 10 μM MSI-1436 for 2 hours. Total RNA was extracted from cells using TRIzol reagent (Thermo Scientific, Cat# 15596018). Chloroform (200 μl) was added to 1 mL TRIzol and incubated at room temperature for 10 minutes. After centrifugation at 10,000 x g for 15 minutes in the cold, the aqueous phase was taken, mixed with an equal volume of isopropanol and supplemented with 0.5 μl glycogen, to increase RNA recovery. Samples were kept at -80°C for 30 min, followed by centrifugation at 10,000 x g for 10 minutes in the cold. The RNA pellet was washed once with 80% Ethanol and dissolved in DEPC-treated water. Poly-A-tailed mRNA was isolated with NEBNext Poly(A) mRNA Magnetic Isolation Module (NEB, E7490). The RNA-seq library was prepared with NEBNext Ultra™ II RNA Library Prep Kit (NET, E7770) for Illumina

sequencing, following the manufacturer's instructions. Samples were pooled together and sequenced on NextSeq with Single Read 75 bases.

RNA-seq analysis. The sequencing reads were aligned to customized mm10 gtf-containing protein coding genes by using salmon 1.0.0 with default setting. Expressed genes (TPM > 0.5 in either control or treatment) were subjected to differential gene expression analysis with DESeq2. Genes were then ranked by their log2 fold change and upregulated genes were subjected to g:Profiler for Reactome analysis. (GEO accession: GSE184197)

Flow cytometry of blood. Blood samples were treated with ACK buffer to lyse the red blood cells (RBCs), and washed with Hanks' Balanced Salt solution (HBSS). Cells were incubated, for 30 min on ice, in FACS-sorting buffer (2 mM EDTA, 0.5% heat-inactivated fetal bovine serum (FBS) in PBS) containing neutrophil surface marker antibodies. When only surface markers were to be measured, cells were examined using a BD LSRFortessa DUAL flow cytometer (BD Biosciences) and data were analyzed by FlowJo software (Tree Star). When intracellular staining for myeloperoxidase (MPO) was needed, cells were fixed with 4% PFA for 10 min at room temperature, permeabilized with 0.1% Triton-X100, and blocked with PBS containing 5% donkey serum and 0.1% Triton X-100 for 1 hour. Samples were next incubated at 4°C overnight, washed with PBS, and incubated with Donkey anti-Goat IgG, Alexa 488 (1:400, Invitrogen, A-11055) for 1 hour at room temperature. To determine the levels of surface markers or MPO in the neutrophils, the scatter plots were gated on Ly6G^{high} population, and the MFI of surface markers or MPO were calculated.

**Antibody**	**Clone**	**Species**	**Manufacturer**
Ly-6G-FITC	1A8	Mouse	eBioscience
Ly-6G-Alexa Fluor 647	RB6-8C5	Mouse	R&D systems
CD62L-APC	MEL-14	Mouse	BioLegend

CXCR2-PerCP/Cyanine5.5	SA044G4	Mouse	BioLegend
CXCR2-Alexa Fluor 594	242216	Mouse	R&D systems
CD11b-FITC	M1/70	Mouse/Human	BioLegend
CXCR4-PE	2B11	Mouse	BD Biosciences
CXCR4-Alexa Fluor 488	247506	Mouse	R&D systems
Anti-Myeloperoxidase	Polyclonal	Mouse/Human	R&D systems

Confocal immunofluorescence microscopy. Balb/c mice were injected either with saline or 10mg/kg MSI-1436, and blood was collected by cardiac puncture 2.5 hours after injection. ACK buffer was added to lyse the RBCs. After washing with HBSS, cells were resuspended in RPMI-1640, and plated onto poly-L-lysine coated 8-well μ-slide (Ibidi) at 20,000 to 50,000 cells per well. The cell culture was incubated at 37 °C for 30min to allow attachment of neutrophils. To stain for MPO, the cells were first fixed with 4% PFA for 10min, washed with PBS, blocked and permeabilized with 5% donkey serum, 0.1% Triton X-100 in PBS for 1 hour at room temperature, and incubated with anti-MPO (1:300, R&D systems, AF3667) overnight at 4°C. Next day, cells were washed with PBS, incubated with Donkey anti-Goat IgG, Alexa 488 (1:400) and DAPI (Abcam, ab228549) for 1 hour at room temperature, washed with PBS and then Ibidi mounting medium was added (Ibidi, 50001). Images were acquired using a Zeiss LSM 780 confocal laser scanning microscope with a Zeiss Plan-Apochromat 63x/1.4 NA Oil DIC M27 objective lens and Zen black acquisition software. The intensity mean of MPO per cell was quantified using the Surface tool in the Surpass and 3D View window of Imaris 3D/4D Visualization and Analysis Software (Bitplane, Version 9.6.1, Oxford Instruments). All the images were processed with the consistent parameter settings of thresholding at absolute intensity 200, and seed density diameter of 6.5 μm to split touching objects.

Neutrophil Extracellular Trap formation with mouse neutrophils. For the *ex vivo* study, mice were injected intraperitoneally with either saline, 2mg/kg MSI-1436 or 10 mg/kg MSI-1436. Peripheral blood was collected through cheek bleeding 2.5 hours after compound administration, and 50 μl blood was required for each well. For the *in vitro* study, blood was obtained from untreated mice. Red Blood Cells (RBCs) were removed with ACK buffer, and the leukocytes were plated onto poly-L-lysine-coated 8-well μ-slide. After 30 min incubation at 37°C to allow neutrophils to attach, DMSO or PMA (100 nM) was added for ex vivo test; DMSO, PMA, or PMA together with MSI-1436 was added for *in vitro* test. Two hours later, cells were fixed, permeabilized and stained for MPO, citH3 (Abcam, ab5103), DAPI, and were visualized using a Zeiss LSM 780 confocal laser scanning microscope. Quantitation was performed based on triple colocalization of DNA, MPO and citH3, using ImageJ and a custom-made macro, available in FigShare (DOI: 10.6084/m9.figshare.14401958).

Neutrophil Extracellular Trap formation with human neutrophils. Whole blood was taken from three healthy volunteers (20-40-year-old, male and female) with informed consent and approved by the IRB of CSHL (IRB-13-025). The blood was collected into BD Vacutainer K2EDTA tube using venipuncture. After lysing RBCs with ACK buffer, the neutrophils were isolated using Percoll density gradient centrifugation as done in isolation of mouse BM neutrophils. The NETosis stimulation and imaging were performed as with mouse neutrophils.

Whole mount staining. To confirm the presence and abundance of NETs in the lungs of mice after TRALI induction, we performed whole-mount immunostaining and tissue clearing of excised lungs. Mice were subject to TRALI and euthanized with CO_2 30 min after MHC-I antibody injection. Mice were then perfused with 20 ml of saline through the left ventricle of the heart, and the lungs were collected in cold PBS. Afterwards, lungs were fixed at 4 °C overnight in PBS with 4% PFA and 30% sucrose. After three washes of 1 h with PBS at room temperature, tissues were permeabilized in methanol gradients in PBS for 30 min (PBS > 50% MeOH > 80%

MeOH > 100% MeOH). Then, tissues were bleached with Dent's bleach (15% H_2O_2, 16.7% DMSO in MeOH) for 1 h at room temperature, and rehydrated through descending methanol gradients in PBS (80% MeOH > 50% MeOH > PBS). Then tissues were incubated with blocking buffer containing PBS with 0.3% Triton X-100, 0.2% BSA, 5% DMSO, 0.1% azide and 25% FBS overnight at 4 °C with shaking. Afterwards, lungs were stained with antibodies against cit-H3 (Abcam, ab5103), MPO (R&D, AF3667) and CD31 (BioLegend, 102502) for 2 days at 4 °C with shaking. After washing for 24 h in washing buffer (PBS with 0.2% Triton X-100 and 3% NaCl), the tissues were stained with secondary antibodies for 24 h at 4 °C with shaking. After 24 h, tissues were washed for 24 h in washing buffer and were dehydrated in MeOH gradients in dH_20 using glass containers for 30 min in each step (50% MeOH > 70% MeOH > 90% MeOH > 3×100% MeOH). Finally, tissues were submerged for 30 min in MeOH with 50% BABB (benzyl alcohol:benzyl benzoate, 1:2) and afterwards cleared in 100% BABB and imaged on a Leica SP8 X confocal microscopy system. Quantitation was performed with Imaris (Bitplane), using spots on a triple-colocalization channel of DNA, MPO and citH3. Neutrophils were quantified using spots based on MPO signal. Frequency was calculated as the number of NETs / number of neutrophils in the 3D volume.

Administration of AZD-5069. The CXCR2 antagonist AZD-5069 (MedChemExpress, HY-19855) was given to mice orally (100 mg/kg). Before feeding AZD-5069, mice were trained to consume 100 μl 10% sucrose through pipette tips for 3 days. On the day of oral administration of AZD-569, compound was freshly prepared in 10% DMSO, 40% PEG-300, 5% Tween-80, 45% physiological saline (0.9%). AZD-5069 was completely dissolved before adding next solvent. AZD-5069 solution (20 mg/ml) was kept in the 50°C water bath until ready to feed mice.

Immunoblots. After PMA or CXCL12 stimulation, the 6-well plates were place on ice and lysed with RIPA buffer, containing freshly added cOmplete™ EDTA-free protease inhibitors (Roche, 11836153001) and Halt phosphatase inhibitors

(Thermo Fisher Scientific, 78428). Equal amounts of cell lysate were loaded onto SDS-PAGE and transferred to Amersham nitrocellulose membrane (Cytiva). The membranes were blocked in 5% BSA in Tris-buffered saline Tween-20 (TBST), followed by overnight incubation with primary antibodies at 4°C. All the primary antibodies were purchased from Cell Signaling Technology (AKT, 9272 and 4691; pAKT T308, 2965; pAKT S473, 9271; Rps6, 2217; pRps6 S235/236, 4858; ERK1/2, 9102; pERK1/2 T202/Y204, 9101; HSP90, 4877). After washing with TBST, secondary antibodies (Peroxidase AffiniPure Goat anti-Rabbit IgG (H+L), Jackson ImmunoResearch Laboratories) were applied and following a TBST wash, the blots were developed with Pierce ECL Western Blotting Substrate (Thermo Fisher Scientific).

Administration of Palomid 529. Palomid 529 (Selleck Chemicals, S2238) was freshly prepared in a micronized formulation in 8% DMSO, 40% PEG300, 5% Tween80, 47% ddH_2O, in which the solvents were added individually and in the order in which they are listed. Before each injection, the tube was vortexed to retain suspension. The compound was administered IP in doses up to 25 mg/kg.

Chemotaxis assay. Chemotaxis assays were performed using Corning HTS Transwell-24 well permeable supports (6.5 mm diameter, 3 μm pore size, Sigma, CLS3398-2EA) and Corning Ultra-Low attachment plates (Sigma, CLS3473-24EA). Either 600 μl of RPMI-1640 as a negative control, or RPMI-1640 containing 100 ng/ml CXCL12 (R&D systems, 460-SD-010), was added to the lower chambers. The upper chambers were seeded with neutrophils, 200 μl at 5 X 10^6 cells/ml. For the treatment groups, the neutrophils were pre-treated with the indicated concentration of PTP1B inhibitors for 30 min, and then loaded into the upper chambers. After 1-2 hours incubation at 37°C, 5% CO_2, EDTA was added to the lower chambers to a final concentration of 10 mM, for 10 min, to detach the cells. The number of neutrophils in the lower chamber were counted using Guava easyCyte.

Statistical analysis. Statistical analyses were performed with Prism software (GraphPad Software). All data are presented as mean ± standard error of the mean (SEM). Comparison between two groups were analyzed by unpaired two tailed t-test. Comparison for more than two groups were analyzed using one-way ANOVA followed by indicated post-hoc test. Statistical significance of Kaplan-Meier curves was determined by Log-rank (Mantel-Cox) test.

Study approval. All the mouse studies were performed in accordance with procedures approved by the IACUC at CSHL and NIH Guide for Care and Use of Laboratory Animals. Whole blood from healthy volunteers was collected with informed consent and approved by the IRB of CSHL (IRB-13-025).

Supplemental References

1. J. Cools-Lartigue *et al.*, Neutrophil extracellular traps sequester circulating tumor cells and promote metastasis. *The Journal of clinical investigation* **123**, 3446-3458 (2013).

2. D. W. Siemsen, I. A. Schepetkin, L. N. Kirpotina, B. Lei, M. T. Quinn, "Neutrophil isolation from nonhuman species" in Neutrophil Methods and Protocols. (Springer, 2007), pp. 21-34.

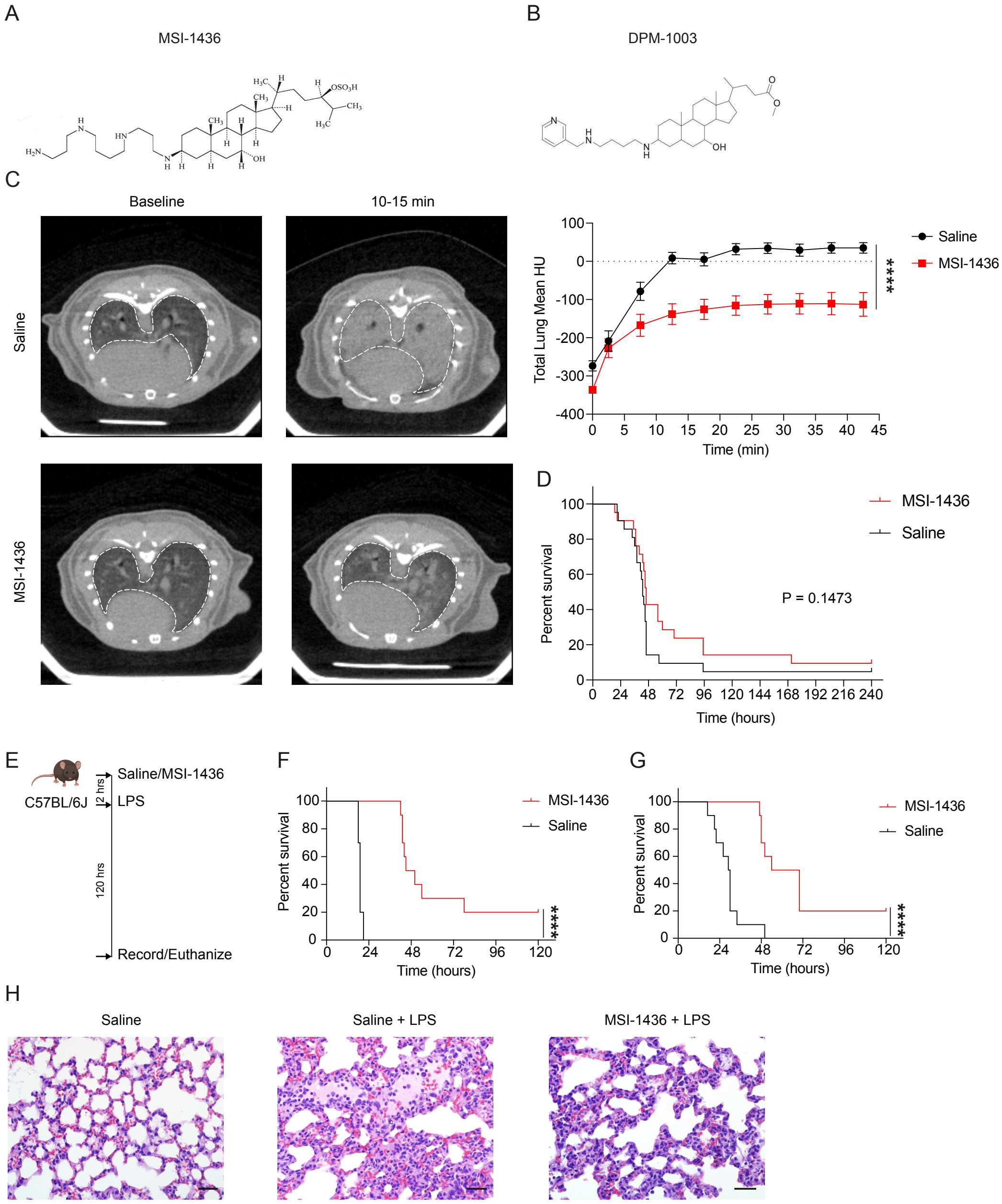

Supplemental Figure 1, related to Figure 1. The effect of MSI-1436 on lung edema accumulation in the TRALI model, and survival in CLP- and LPS-induced sepsis models.

(A-B) The structural formulae of PTP1B inhibitors MSI-1436 (A) and DPM-1003 (B).

(C) Left panel: the representative transverse CT images from TRALI mice treated with either saline or MSI-1436. Dashed lines indicate the boundaries of the total

lung ROI. Right panel: the mean lung radiodensity measured by longitudinal CT scans in two experimental groups (n=7-8 mice per group). HU 0 = density of water and -1,000 = density of air.

(D) The survival curves of CLP-induced sepsis model treated either with saline or 5 mg/kg MSI-1436, six hours after surgery (n=21 mice per group).

(E) Schematic illustration of LPS-induced sepsis model.

(F) The survival curves of 30 mg/kg LPS challenged sepsis mice treated with either MSI-1436 or saline (n=10 mice, two independent biological repeats).

(G) The survival curves of 15 mg/kg LPS challenged sepsis mice treated with either MSI-1436 or saline (n=10 mice, two independent biological repeats).

(H) Representative H&E staining images of lung tissues harvested from mice treated with saline only, saline 2 hours prior to LPS (15 mg/kg), and MSI-1436 2 hours prior to LPS (15 mg/kg) administration.

Statistical analysis for C by two-way ANOVA; D, F and G by Log-rank (Mantel-Cox) test; ****p<0.0001.

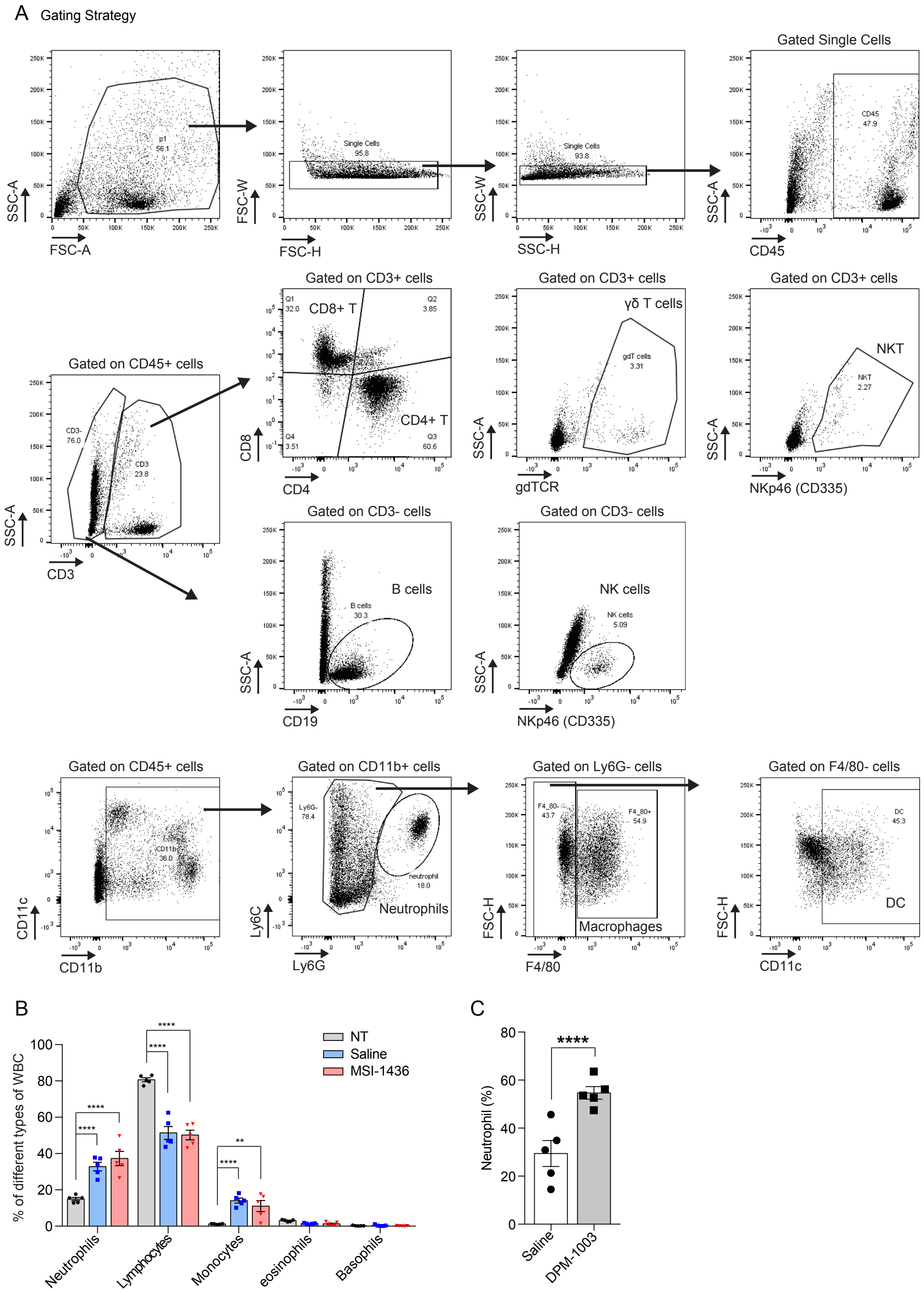

Supplemental Figure 2, related to Figure 2. Characterization of immune cells following MSI-1436 treatment.

(A) The gating strategy used to identify the nine immune cell populations from the single cell suspension of lung tissue.

(B) The percentages of WBC subsets measured in the circulating blood collected from either NT or TRALI mice. For TRALI mice, the blood was collected 30 min after anti-MHC I antibody injection, and pretreated with either saline or 10 mg/kg MSI-1436 (n=5).

(C) Percentage of neutrophils (relative to total WBCs) in peripheral blood from mice treated with saline, or 20 mg/kg DPM-1003 for 2.5 hours (n=5).

Data are presented as mean ± SEM. Statistical analysis for B by two-way ANOVA with Tukey's multiple comparisons test; for C by two-tailed student's t-test; $^{**}p<0.01$, $^{****}p<0.0001$.

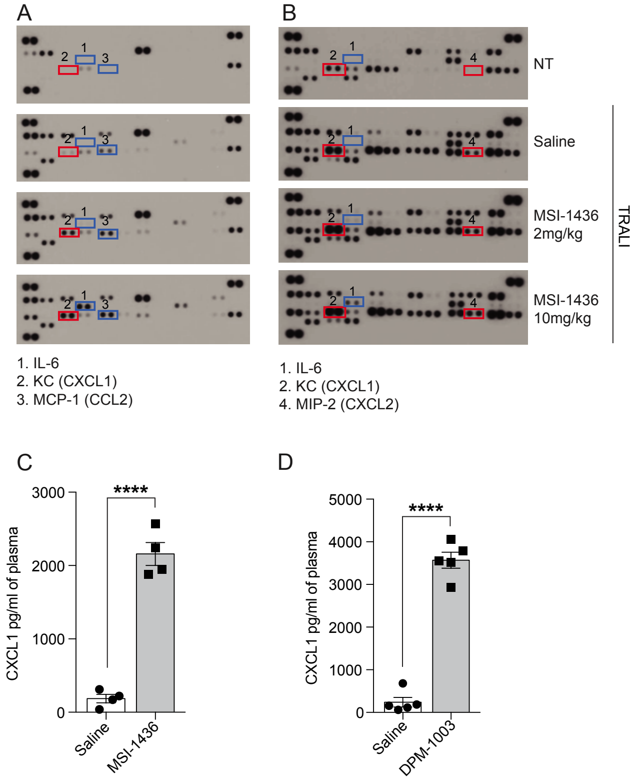

Supplemental Figure 3, related to Figure 2. Characterization of cytokine levels following PTP1B inhibitor treatment.

(A-B) Cytokine arrays generated from serum (A) or lung tissue (B) collected from NT or TRALI mice treated with saline or MSI-1436. Each group contained an equal amount of serum or lung tissue homogenate pooled from 5 mice.

(C-D) CXCL1 was measured in the plasma of mice treated with saline, 10 mg/kg MSI-1436 (C) or 20 mg/kg DPM-1003 (D).

Data are presented as mean ± SEM, where n=5 mice per group. Statistical analysis for C and D by two-tailed student's t-test; ****p<0.0001.

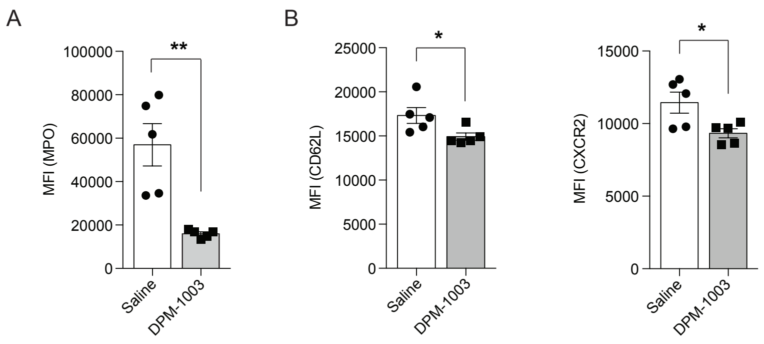

Supplemental Figure 4, related to Figure 3. Treatment with DPM-1003 promoted neutrophil aging *in vivo*.

(A) The flow cytometry analysis of MPO in the neutrophils following treatment with saline or DPM-1003 for 2.5 hours. MPO was quantified by MFI.

(B) The flow cytometry analysis of fresh neutrophil markers CD62L and CXCR2 following treatment with saline or DPM-1003 for 2.5 hours, quantified as MFI.

Data are presented as mean ± SEM. Statistical analysis for A and B by two-tailed student's t-test; $^*p<0.05$, $^{**}p<0.01$.

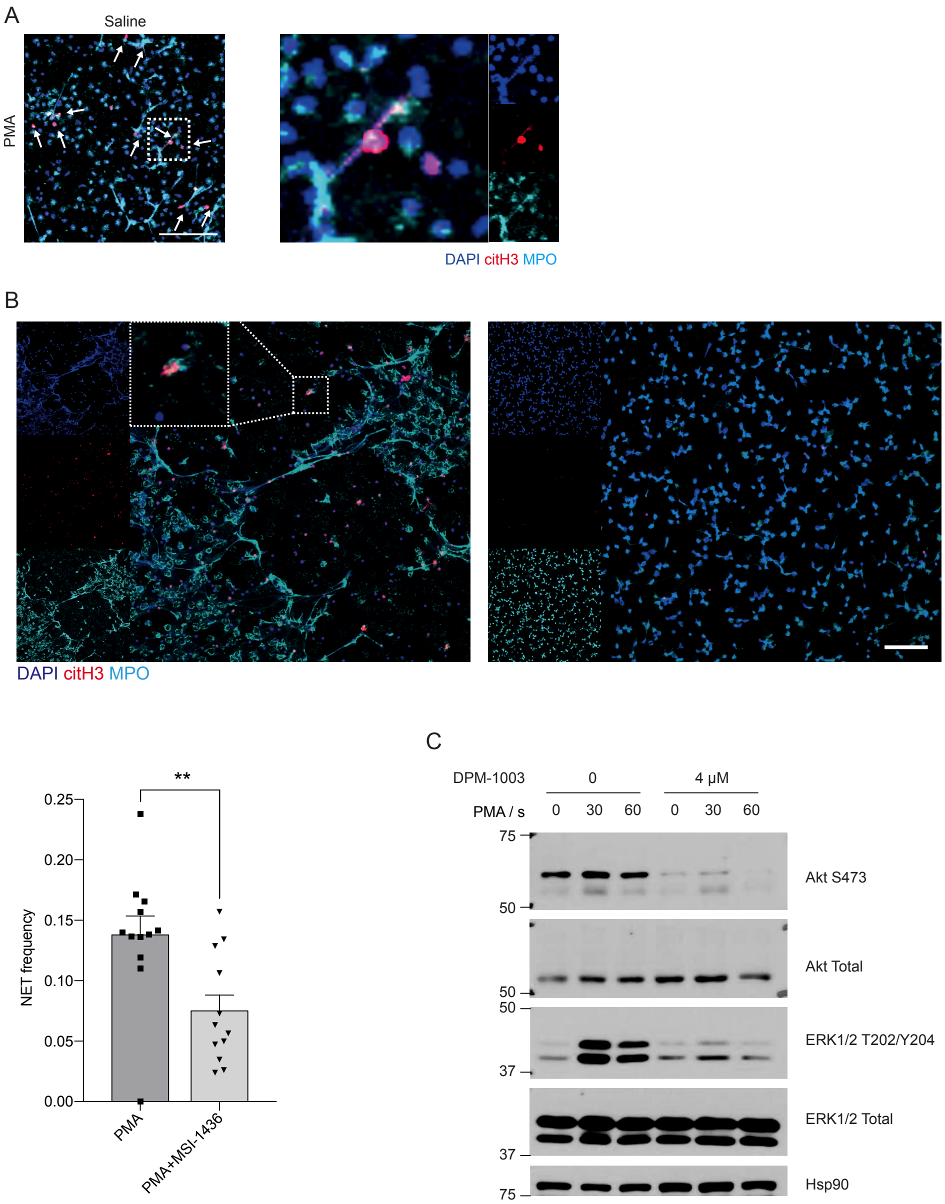

Supplemental Figure 5, related to Figure 4. PTP1B inhibitors reduced PMA-mediated NETosis and Akt signaling in the neutrophils.

(A) The higher magnification detailed image from Figure 4A lower left panel with three individual channels to show colocalization of DAPI, citH3 and MPO.

(B) Representative confocal images and associated quantification, showing the NET frequency in response to PMA (left panels) or MSI-1436+PMA (right panel) stimulation of human primary neutrophils. Scale bars: 100 μm, n=3 healthy volunteers. Data are presented as mean ± SEM. Statistical analysis by two-tailed student's t-test, **p<0.01.

(C) Neutrophils isolated from bone marrow (BM) were stimulated with 100 nM PMA for the indicated time. Immunoblot analysis showing DPM-1003 attenuated Akt and Erk1/2 phosphorylation. The numbers on the left indicate molecular weight standards.

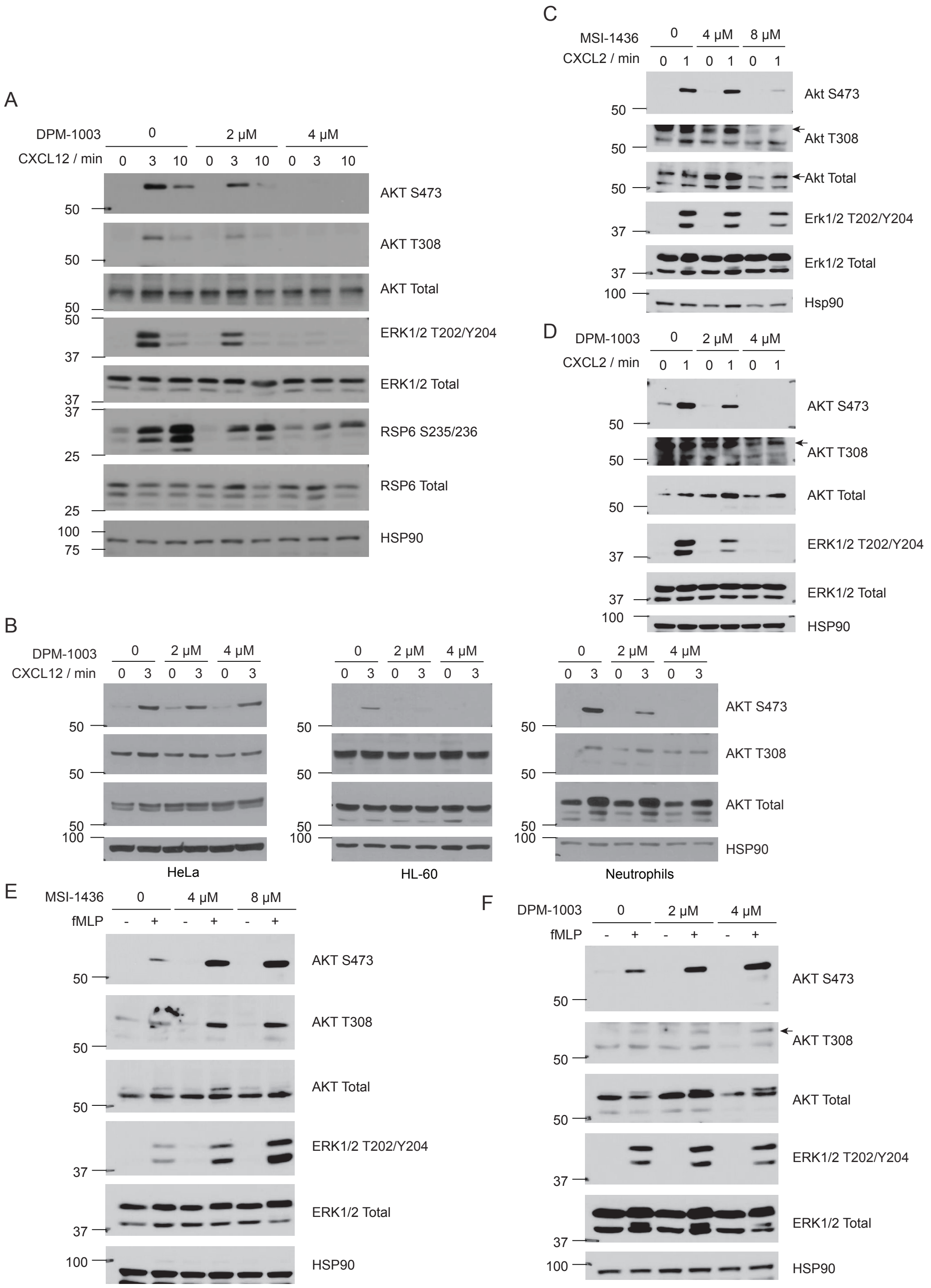
A
DPM-1003
CXCL12 / min
AKT S473
AKT T308
AKT Total
ERK1/2 T202/Y204
ERK1/2 Total
RSP6 S235/236
RSP6 Total
HSP90
B
HeLa
HL-60
Neutrophils
C
MSI-1436
CXCL2 / min
Akt S473
Akt T308
Akt Total
Erk1/2 T202/Y204
Erk1/2 Total
Hsp90
D
E
fMLP
F

Supplemental Figure 6, related to Figure 5. The effect of PTP1B inhibitors on AKT signaling.

(A) Immunoblot analyses of DPM-1003 treated neutrophils showing dose-dependent inhibitions of CXCR4-mediated AKT signaling.

(B) Immunoblot analyses of the effect of DPM-1003 on AKT signaling in HeLa, HL-60, and mouse primary neutrophils upon CXCL12 stimulation.

(C-D) Immunoblot analyses showing the decrease of CXCL2-stimulated AKT and ERK1/2 phosphorylation upon MSI-1436 (C) and DPM-1003 (D) treatment.

(E-F) Immunoblot analyses showing the increase of fMLP stimulated AKT and ERK1/2 signaling upon MSI-1436 (E) and DPM-1003 (F) treatment. The numbers on the left indicate molecular weight standards.

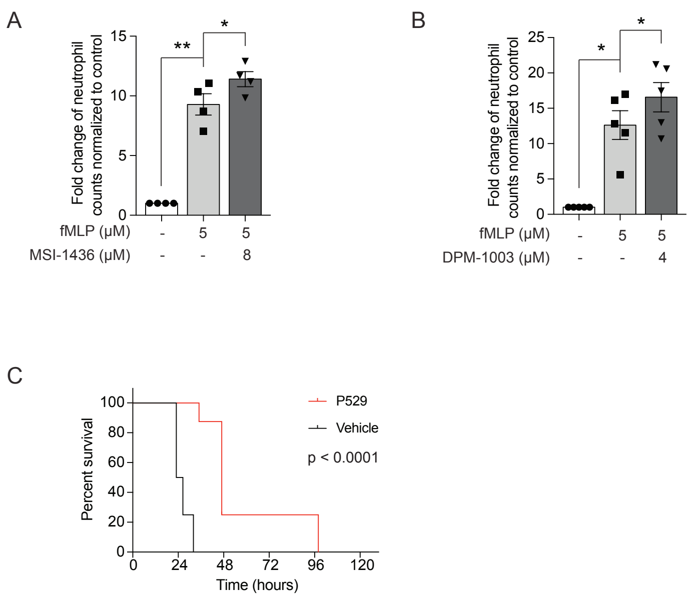

Supplemental Figure 7, related to Figure 6. Neutrophil migration regulated by PTP1B inhibitors and the survival of LPS-induced sepsis prolonged by P529 treatment.

(A-B) The impact of PTP1B inhibitors MSI-1436 (E) and DPM-1003 (F) on neutrophil migration towards fMLP examined using Transwell assays. n=4-5 independent biological repeats.

(C) P529 prolonged survival in LPS-induced sepsis model.

Data are presented as mean ± SEM. Statistical analysis for A and B by one-way ANOVA with Tukey's multiple comparisons test; for C by Log-rank (Mantel-Cox) test**.** *P<0.05, **p<0.01.

Supplemental Tables

Supplemental Table 1. Peripheral blood analyses after saline, MSI-1436 or DPM-1003 treatment.

	Saline	MSI-1436	DPM-1003
**Hematologic parameters**			
Hemoglobin level, g/dL	15.8±0.2	15.3±0.4	16.8±0.2
Hematocrit (%)	50.6±0.5	48.6±1.3	52.3±0.5 *
WBC count, $X10^9/L$	10.0±0.8	12.2±0.7	11.9±0.7
RBC count, $X10^{12}/L$	10.9±0.1	10.6±0.3	11.5±0.1 *
Platelet count, $X10^9/L$	964.0±29.7	811.4±48.4 *	813.2±85.7
**WBC differential count**			
Neutrophils, $X10^9/L$	2.0±0.3	6.2±0.5 ****	6.1±0.3 ****
Monocytes, $X10^9/L$	0.19±0.05	0.73±0.16 *	0.43±0.05 *
Basophils, $X10^9/L$	0.004±0.002	0.016±0.006	0.016±0.004
Eosinophils, $X10^9/L$	0.094±0.015	0.052±0.005 *	0.034±0.007 ***
Lymphocytes, $X10^9/L$	7.7±0.5	5.1±0.2 **	5.3±0.5

Data are presented as mean±SEM (n=5 for each group). Statistical analysis by two-tailed student's t-test to compare PTP1B inhibitor treatment and saline; *P<0.05, *** p<0.001, ****p<0.0001.

Supplemental Table 2. Reactome pathway enrichment analysis of up-regulated genes upon MSI-1436 treatment.

**Reactome**		
**Term name**	**Term ID**	**Padj**
Neutrophil degranulation	REAC:R-MMU-6798695	4.76E-06
Immune system	REAC:R-MMU-168256	3.57E-04
Innate immune system	REAC:R-MMU-168249	2.37E-03
Formyl peptide receptors bind formyl peptides and many other ligands	REAC:R-MMU-444473	1.27E-02

Supplemental Movie Legends

Supplemental Movie 1: Time series CT scan images of chest from TRALI mice treated with saline.

Representative video of longitudinal CT scans from one of seven mice treated with saline 2 hours before anti-MHC-I antibody injection. Video shows baseline to 15 minutes post-antibody injection (endpoint).

Supplemental Movie 2: Time series CT scan images of chest from TRALI mice treated with MSI-1436.

Representative video of longitudinal CT scans from one of eight mice treated with MSI-1436 2 hours before anti-MHC-I antibody injection. Video shows baseline to 45 minutes post-antibody injection.

Supplemental Movie 3: Whole-mount immunostaining of cleared lungs showing NET formation in the lungs from mice treated with saline 2 hours before TRALI induction.

Representative video of four independently cleared lungs is shown. Lungs were harvested 30 min after MHC-I antibody injection and stained for MPO (neutrophils, cyan), DNA (blue), citH3 (red) and CD31 (white). NETs were defined as triple-positive MPO-DNA-citH3 events.

Supplemental Movie 4: Whole-mount immunostaining of cleared lungs showed reduced number of NETs in the lungs from mice treated with 10 mg/kg MSI-1436 2 hours before TRALI induction.

Representative video of four independently cleared lungs is shown. Lungs were harvested 30 min after MHC-I antibody injection and stained for MPO (neutrophils, cyan), DNA (blue), citH3 (red) and CD31 (white). NETs were defined as triple-positive MPO-DNA-citH3 events.
